# Enhancing subscription fraud detection through ensemble learning the case of Ethio telecom

**DOI:** 10.1038/s41598-026-38790-3

**Published:** 2026-02-09

**Authors:** Esubalew Asmare Desta, Kidus Workineh Azale, Abenet Alazar Hailu, Fikadu Berie Adugna, Alexander Takele Mengesha, Selamawit Fentie Belay, Habtamu Ayenew Asegie, Ayodeji Olalekan Salau

**Affiliations:** 1 Esubalew Asmare Desta: College of Informatics, University of Gondar, Gondar, Ethiopia; 2 Kidus Workineh: College of Informatics, University of Gondar, Gondar, Ethiopia; 3 Abenet Alazar Hailu: College of Informatics, University of Gondar, Gondar, Ethiopia; 4 Fikadu Berie Adugna: College of Informatics, University of Gondar, Gondar, Ethiopia; 5 Alexander Takele Mengesha: College of Informatics, University of Gondar, Gondar, Ethiopia; 6 Selamawit Fentie Belay, Department of Information Science, College of Informatics, University of Gondar, Gondar, Ethiopia; 7 Habtamu Ayalew Asegie, Department of Information Technology, College of Informatics, University of Gondar, Gondar, Ethiopia; 8 Ayodeji Olalekan Salau, Department of Electrical/Electronic and Computer Engineering, Afe Babalola University, Ado-Ekiti, Nigeria; 9 Saveetha School of Engineering, Saveetha Institute of Medical and Technical Sciences, Chennai, Tamil Nadu India

**Keywords:** Subscription fraud, Fraud detection, Ensemble learning, Adaptive Learning6, Decision tree, Adaptive random forest, Stacking, Call detail records (CDRs), Hyperparameter tuning, Engineering, Mathematics and computing

## Abstract

Telecommunication companies globally face the critical challenge of subscription fraud, which threatens both financial stability and national security. This research addresses this issue by developing an advanced fraud detection model specifically for Ethio Telecom. The model utilizes Ensemble and Adaptive Learning techniques to enhance detection accuracy by combining multiple classifiers. The study used a dataset of 1,000,000 Call Detail Records (CDRs) collected over two months known for increased fraudulent activity3. After filtering out irrelevant data and aggregating multiple call records per subscriber, the dataset was refined to 349,164 records. Initially, 16 features were analyzed, with four excluded for lacking relevance. The remaining 11 features, excluding the target variable, underwent preprocessing including data cleaning, transformation, and balancing4. Feature selection, utilizing Correlation Matrix and Random Forest importance analysis, led to the removal of four additional features, resulting in a final set of 8 key features, including INT_DIALLED, RATIO_INT_TOTAL, and RATIO_UNIQUE_TOTAL4. Three individual models, namely Decision Tree (DT), Logistic Regression (LR), and Artificial Neural Network (ANN), were implemented alongside ensemble methods such as Bagging, Boosting, Stacking, and Voting, and adaptive models like Hoeffding Tree and Adaptive Random Forest45. The findings of this research recommend Stacking and Adaptive Random Forest (ARF) as robust tools for subscription fraud detection.

## Introduction

Telecommunications in Ethiopia began in 1894 with the Ethiopian Telecommunications Corporation (ETC), renamed Ethio Telecom in 2011. As the country’s main provider, it offers phone, internet, and mobile money services, serving 72 million users in 2023/24, including 69.5 million mobile subscribers. Despite its growth, advancing technology and rising customer demands have heightened fraud risks in the sector^1^. Telecommunications fraud targets subscription, account, and network systems, causing financial losses and security risks. Subscription fraud, using fake or stolen identities for unpaid services, is a major issue for Ethio Telecom, leading to high uncollectable revenue. Subscription fraud is a global threat to telecom providers, with the CFCA reporting a 12% rise in 2023, causing $38.95 billion in losses about 2.5% of global telecom revenues^2,3^.

Subscription fraud is the largest contributor to telecom losses, exceeding $7 billion annually and accounting for 31.6% of top fraud types in 2013, according to CFCA. Ethio Telecom alone loses about $1 billion yearly to fraud, with subscription fraud representing 40% of total losses, exposing weaknesses in its rule-based fraud management system. These figures highlight the urgent need for stronger safeguards to protect revenues and customers. To address this, Ethio Telecom has established Fraud Management (FM) and Revenue Assurance (RA) units equipped with a Fraud Management System (FMS) and fraud detection experts^4^.

Fraud detection initially relied on manual audits and expert-defined rule-based systems that lacked flexibility against complex scams. The advent of machine learning (ML) transformed the field by enabling automated, scalable, and real-time fraud detection through data-driven pattern and anomaly analysis. ML algorithms such as ANN, Decision Trees, SVM, Naive Bayes, Random Forest, and K-NN improve accuracy by learning from new data, while ensemble learning combining multiple classifiers enhances the robustness and efficiency of modern fraud detection systems^2,5,6^.

In spite of the developments, the Ethiopian telecommunications sector is under great threats from fraudulence that can compromise its sustainability and growth. The absence of modern fraud detection solutions, including the static nature of rule-based systems and the use of single models as reported by earlier Ethiopian studies using tools such as Weka, highlights the importance of developing a more mature and dynamic solution. Ensemble learning offers a fantastic exit strategy by combining heterogeneous models to build an augmented perception of patterns and anomalies in subscriber usage records, i.e., Call Detail Records (CDR), thereby enriching accuracy and efficiency and ultimately saving telecommunication operators a substantial amount of monetary losses^2^.

### Problem statements

The telecommunications industry in Ethiopia is undergoing unprecedented growth, but the persistent challenge of telecom fraud poses a significant threat to its sustainability and expansion. Ethio telecom, a major player in the market, faces an annual revenue loss of 1 billion dollars due to fraud, emphasizing the urgency to address this critical issue^7^. Subscription Fraud (SF) is a major contributor to revenue loss, constituting 40% of the overall losses and highlighting the limitations of the current rule-based fraud management system^8^.

Previous research efforts in Ethiopia have primarily relied on single models and the Weka tool for fraud detection^4,9–11^, indicating a gap in exploring more advanced and robust approaches. The limitations of single models and the static nature of rule-based systems underscore the need for a more sophisticated and adaptive solution. Various data mining techniques, including rule mining, clustering, Bayesian networks, neural networks, and decision trees, are employed for fraud detection in the telecommunications industry^7^. This research is important because it takes another way to detect fraud by using the power of ensemble learning and adaptive learning for subscription fraud detection.

Ensemble learning, particularly its application in machine learning, is considered superior in this context for several reasons. Unlike single models, ensemble learning combines the strengths of multiple models, mitigating the weaknesses of individual models. This approach enhances the overall predictive performance, generalization, and robustness of the fraud detection system. In the face of evolving fraud tactics, ensemble learning’s ability to adapt and learn from diverse models makes it an ideal candidate for addressing the dynamic challenges posed by fraudsters^12,13^.

Furthermore, ensemble learning can effectively handle the complex and vast dataset generated by the telecommunications network^11^. The integration of diverse models in an ensemble provides a more comprehensive understanding of patterns and anomalies in subscriber usage data, specifically Call Detail Records (CDR). This holistic approach enhances the accuracy and efficiency of fraud detection, ultimately saving telecommunication operators substantial financial losses.

### The contribitions of study

This study provides a comprehensive exploration of subscription fraud detection in the Ethiopian telecommunications sector using advanced ensemble and adaptive machine learning models. A major contribution lies in addressing underexplored phenomena namely, the dynamic and context-specific nature of fraud in Ethiopia, the limitations of traditional detection methods, and the integration of ethical considerations such as data privacy and security.

The research investigates the previously underutilized potential of ensemble models like *Stacking* and adaptive models like *Adaptive Random Forest (ARF),* highlighting their superior performance, 99.3% and 99.2% accuracy respectively in identifying fraud. This contrasts significantly with past Ethiopian studies that relied on single models and static tools such as Weka. By showing how adaptive algorithms dynamically update with evolving fraud patterns, the study provides a data-driven solution to an ever-changing threat landscape.

Furthermore, the study uncovers *context-specific indicators* of fraud, such as INT_DIALLED, RATIO_INT_TOTAL, and RATIO_UNIQUE_TOTAL, derived from Call Detail Records (CDRs). These engineered features explain fraudulent behavior more effectively than generic variables, thereby extending fraud detection theory with domain-relevant evidence.

A particularly underexplored phenomenon addressed is *real-time feature selection*. The research utilizes *Online Streaming Feature Selection (OSFS)* to maintain model relevance as data patterns shift, ensuring sustained accuracy without full retraining—an area neglected in previous work.

Importantly, the study also *raises the ethical dimension* of fraud detection by discussing data anonymization, audit trails, and restricted access, implicitly acknowledging data privacy as a critical but overlooked component in technical fraud research.

In bridging the gap between theory and practice, the study proposes a *model architecture* that can be directly applied by Ethio Telecom. It also offers *strategic recommendations* for future implementation, such as using Apache Kafka for real-time monitoring and expanding the models to other fraud types.

Overall, the research not only introduces high-performing detection techniques but also explains why they work better in Ethiopia’s context making it a foundational study in both technological advancement and contextual understanding of subscription fraud.

In general the summary of key Innovations of the Study):

- *Ensemble and Adaptive Models:* Introduces Stacking and Adaptive Random Forest (ARF) models that outperform single-model approaches, achieving up to 99.3% accuracy and adapting dynamically to evolving fraud patterns.
- *Context-Specific Fraud Features:* Develops telecom-specific indicators such as *INT_DIALLED*, *RATIO_INT_TOTAL*, and *RATIO_UNIQUE_TOTAL* from Call Detail Records (*CDRs*), enhancing the precision of fraud detection in Ethiopia’s unique context.
- *Real-Time Adaptability:* Implements *Online Streaming Feature Selection* (*OSFS*) to maintain model accuracy as data changes, eliminating the need for frequent retraining.

### Ethical and practical integration:

- Incorporates *data privacy*, *anonymization*, *and audit trail mechanisms*, ensuring secure and responsible fraud detection.
- *Operational Impact:* Proposes a deployable model architecture tailored for *Ethio Telecom*, with recommendations for *real***-***time monitoring* using tools like *Apache Kafka* and potential expansion to other fraud types.

In essence, the study’s originality lies in combining adaptive ensemble learning with domain-specific and ethically grounded innovations to create a scalable, high-performing fraud detection framework for Ethiopia’s telecom sector.

## Literature review

This section reviews global and local literature on telecommunication fraud, covering detection and prevention techniques, algorithms, data processing methods, and predictive model development. It also identifies research gaps that need further exploration and improvement.

### Fraud in the telecommunication industry

Telecom fraud involves various illicit activities primarily driven by the intention of obtaining unauthorized services or generating illicit revenue. Within the telecom industry, fraud specifically targets areas such as subscription processes, account management, and communication networks. Subscription fraud, which involves using stolen or fabricated identities to sign up for services without intending to pay, is prevalent, particularly on GSM networks^14^. It often serves as a gateway to other forms of telecom fraud and is considered one of the most harmful non-technical fraud types. Other types of fraud mentioned in the literature include roaming fraud, where services are used outside the home network without payment, PBX fraud involving unauthorized calls through a company’s switch station5, and cloning fraud by duplicating a legitimate mobile phone’s identity. Fraudulent activities can be driven by revenue generation, such as selling discounted services obtained fraudulently, or non-revenue motivations like avoiding payment or circumventing system security. This constantly evolving challenge necessitates continuous updates and adaptations in fraud detection methodologies^14^.

Machine learning techniques are crucial in enabling systems to learn and integrate knowledge from large-scale data observations8. Supervised learning, which uses labeled datasets, is a foundational approach in machine learning for classification problems^15^. Various supervised learning algorithms, such as Artificial Neural Networks (ANN), Decision Trees, and Logistic Regression, are used in fraud detection, but they face challenges with imbalanced datasets common in this domain^6^,^16^.

### Challenges in detecting telecom fraud

Detecting telecom fraud is an inherently difficult and dynamic task due to the ever-evolving nature of fraud tactics. As detection methods improve, fraudsters adapt by changing their strategies, continually challenging the efficacy of anti-fraud measures. The perpetration of telecom fraud is often orchestrated by organized criminal gangs, professional hackers, and even the service providers’ own employees, adding layers of complexity to the issue^17^.

The widespread availability of sophisticated hacking tools on the internet has democratized the means of committing telecom fraud, enabling virtually anyone with specific technical skills or tools to engage in such activities. The primary motivation behind telecom fraud is revenue generation, such as selling fraudulently obtained telephone services at reduced rates. However, non-revenue driven motivations also exist, including the desire to bypass or minimize payment for services used or to demonstrate the capability to circumvent a provider’s system security. This multifaceted challenge requires continuous updates and adaptations in fraud detection methodologies to effectively combat these evolving threats^17^.

### Review of related works

Previous research efforts in Ethiopia, as evidenced by studies that primarily relied on single models and tools like Weka, indicate a gap in exploring more advanced and robust approaches. The reliance on single models and static rule-based systems highlights the need for more sophisticated and adaptive solutions.

In recent years, several studies have explored different machine learning approaches to subscription fraud detection. The researcher *Rediet W*^18^*.* developed a subscription fraud detection model for Ethio Telecom using a deep learning approach (CNN & DNN) on three months of CDR data, achieving 99.14% accuracy. In this study, the proposed model lacks adaptability, used few features from CDR data, did not consult domain experts, and the attribute selection method was manual and unclear. The researcher *Derebe T*^19^*.* analyzed which machine learning algorithm performs better for detecting subscription fraud using J48, SVM, and ANN on two months of CDR data, achieving accuracies of *99.3%, 97.5%, and 96%* respectively. The study has limited real-world application, lacks adaptability, reports a higher false positive rate, and did not explore broader methodologies like ensemble learning. The research done by *Getahun W*^4^*.* built a predictive model using a deep autoencoder (CNN-LSTM auto encoder) to detect subscription fraud in Ethio Telecom using three months of CDR data, achieving *98.95% accuracy.* In this study, the proposed model has high computational complexity, lacks adaptability, and omits cost analysis of false positives and false negatives. The author *Alemeshet G *^11^. investigated ensemble methods (Boosting using J48) for predicting voice traffic termination fraud using two months of CDR data, achieving *96.73% accuracy*. This study was focused on voice termination fraud, the algorithm selection was not justified, and it lacks adaptability and generalizability, and ignores the cost analysis of incorrect classifications. Research on voice traffic termination fraud using Boosting with J48 focused only on that specific type of fraud, lacked justification for algorithm selection, lacked adaptability and generalizability, and ignored the cost of incorrect classifications. Hailemeskel G/Tsad^10^ constructed a subscription fraud detection model using machine learning algorithms (RF, ANN, and SVM) on postpaid and prepaid CDR data over two months, achieving *99.46% accuracy (using RF).* In this study, the misclassifications were not fully addressed, it lacks adaptive learning capabilities, and has higher false negatives. The study done by authors *Pablo *et al.^20^, aimed to prevent subscription fraud in fixed telecommunications using fuzzy rules and neural networks on CDR data from 47,000 subscribers, achieving *96.78% accuracy*. The study is limited to fixed telecommunications, involves subjectivity in manual classification, risks becoming outdated with evolving fraud patterns, and is prone to false positives. The researhcer *Ledisi *et al.^21^ designed and implemented a subscription fraud detection system using Artificial Neural Networks, achieving *85.7% accuracy*. The limitation os the study, the performance was measured under specific conditions, the accuracy is suboptimal, generalizability was not explored, and scalability was not extensively discussed. The researcher *Tesfaye H *^9^*.* developed a model for detecting and predicting subscription fraud in Ethiopia using data mining techniques (RF, J48) on six months of CDR data, achieving *99.9251% accuracy (using RF).* The study is limited to subscription fraud in Ethio Telecom, has limited algorithm selection, includes minimal discussion on ethical issues in data mining, and features are not discussed.

Collectively, these studies highlight the importance of leveraging advanced techniques, such as machine learning, deep autoencoder methods, and fuzzy rules with neural networks, to enhance subscription fraud detection. However, the varying contexts, datasets, and methodologies employed indicate both progress and challenges in developing robust and generalizable fraud detection systems in the dynamic telecommunications sector. The Table 1 in below provides a summary of the related work.

**Table 1:** A summary of the related work.

No	Author’s Name	Objective	Datasets	Algorithm used	Accuracy	Limitations
1	Rediet W.^18^	Develop a subscription fraud detection model for Ethio Telecom using a deep learning approach	CDR (three months)	CNN & DNN	99.14%Using DNN	Lacks adaptability: Features collected are few from CDR & doesn’t consult domain experts, Attribute selection method is manual & not clearlyjustified
2	Derebe T.^22^	Analyze which Machine learning algorithm perform better for detecting subscription fraud	CDR (two months)	J48, SVM, ANN	99.3%,97.5%and 96%	Limited real-world application, lacks adaptability, higher false positive rate, broader methodologies like ensemble learning not explored
*3*	Getahun W.^4^	Build a predictive model using deep auto encoder to detect subscription fraud in case of Ethiotelecom	CDR(three months)	CNN-LTSM auto encoder	98.95%	High computational complexity, lacks adaptability, and omits cost analysis of false positives and false negatives
*4*	Alemeshet G^11^	Investigate ensemble methods for predicting voice traffic termination fraud	CDR(two months)	Ensemble learning (Boosting using J48)	96.73%	Focused on voice termination fraud, Algorithm selection not justified, lacks adaptability and generalizability, and ignores analysis of cost ofincorrect classifications
*5*	Hailemeskel G/Tsadik^10^	Construct a subscription fraud detection model using machine learning on postpaid and prepaid CDR data	CDR (two months)	RF, ANNandSVM	99.46%(using RF)	Misclassifications not fully addressed, lacks adaptive learning, and has higherfalse negatives
*6*	Pablo et al*.*^20^	To prevent subscription fraud in fixed telecommunications with high impact on long distance carriers	CDR (47,000subscribers)	Fuzzy rules and neural networks	96.78%	Limited to fixed telecommunications; subjectivity in manual classification; risks becoming outdated with evolving fraud patterns; prone to false positives
*7*	Ledisi et al*.*^21^	Design and implement a subscription fraud detection system using ANN	–	ANN	85.7%	Performance measured under specific conditions; accuracy is suboptimal; generalizability not explored; scalability not extensively discussed
*8*	Tesfaye H.^9^	Develop a model for detecting and predicting subscription fraud in Ethiopia’s mobile communication services using data mining techniques	CDR (six months data)	RF,J48	99.9251%(using RF)	Limited to subscription fraud in Ethio Telecom; limited algorithm selection; minimal discussion on ethical issues in data mining, features are not discussed

#### Identified research gaps

In the rapidly evolving field of telecommunications, subscription fraud remains a significant challenge, requiring more advanced detection strategies. Despite progress in machine learning, several key areas remain inadequately addressed, particularly in the context of dynamic environments like Ethiopian telecommunications. This section highlights the critical research gaps my study aims to fill, focusing on adaptive learning capabilities, data privacy and security, and the integration of advanced ensemble learning models.

## Gap in adaptive learning capabilities

Current research often overlooks the adaptive learning capabilities of fraud detection systems, particularly in environments characterized by rapidly evolving fraudulent tactics. Most machine learning models are trained on historical data and may not effectively adapt to new or unknown patterns of fraud without continual retraining. This limitation is critical in the telecom sector, where fraudsters frequently change their strategies to evade detection. Studies such as Abiye^7^ and Abdul et al.^23^ highlight the need for developing models that can dynamically learn and adjust in real-time to new threats, ensuring that fraud detection systems remain effective over time without requiring extensive manual updates. Our study aims to fill this gap by incorporating adaptive online learning capabilities, which will enable the model to continuously update and improve its performance in response to new fraud patterns.

## Insufficient emphasis on privacy and data security

Privacy and data security concerns are paramount in the development and implementation of fraud detection systems, yet they are often underemphasized in current research. Solanki et al.^24^ discuss the necessity of addressing significant privacy issues that arise from using sensitive customer data in fraud detection. Ensuring that these systems protect against fraud while safeguarding customers’ personal information is crucial. This research addresses this gap by focusing on developing fraud detection techniques that maximize data security and privacy. By incorporating robust data anonymization and encryption methods, the proposed system will ensure that sensitive customer information is protected throughout the fraud detection process.

## Implementing advanced ensemble learning models

Most previous research efforts in Ethiopia and similar contexts have primarily relied on single models and static machine learning using tools like Weka for fraud detection^9,10,25^. These approaches have limitations in terms of adaptive predictive performance and robustness. Advanced ensemble learning models, such as bagging, boosting, and stacking, offer potential improvements by combining the strengths of multiple algorithms. However, their application in the context of fraud detection in the Ethiopian telecommunications sector remains underexplored. The research will implement these advanced ensemble learning techniques to enhance the overall predictive performance, generalization, and robustness of the fraud detection system. This approach aims to develop a more sophisticated, adaptive, and effective system capable of handling the complexities and dynamics of telecom fraud.

This research addresses these identified gaps by employing ensemble and adaptive learning techniques for subscription fraud detection at Ethio Telecom. By using advanced methods like Stacking and Adaptive Random Forest (ARF), this study aims to overcome the limitations of previous approaches and provide a more accurate, adaptable, and robust solution. This approach seeks to enhance detection performance and offer a generalizable model capable of combating evolving subscription fraud challenges.

### Research design and methodology

This study adopts a quantitative approach, focusing on the numerical analysis of large datasets to develop a predictive model for subscription fraud detection using ensemble and adaptive learning techniques^26–28^. The research design is experimental, manipulating features from call detail records (CDRs) to classify subscribers as fraudulent or genuine^28^. This approach allows for rigorous testing, control of variables, and comparison of multiple ensembles learning algorithms to identify the most effective model, ensuring the development of a robust fraud detection system.

### Proposed model architecture

The research workflow for the subscription fraud detection system is designed to ensure a systematic and structured approach from data collection to model evaluation. The workflow integrates individual models, ensemble learning techniques and adaptive learning features to enhance the accuracy and robustness of the fraud detection system. Below is a visual architecture of the proposed model workflow:

The above despited workflow illustrates the proposed architecture for subscription fraud detection, starting with the collection and preprocessing of CDR and fraud-related data, which involves data cleaning, balancing, transformation, and feature selection to prepare the dataset for analysis. The data is then split into training, validation, and test sets. The training set is used to train three categories of models: individual models, ensemble models, and adaptive learning models. These models undergo hyperparameter tuning and are subsequently evaluated on the validation and test sets. A comparison of the models is conducted to select the best-performing model, which is then reviewed through expert consultation. If the results are satisfactory, the process ends; if not, the process is revised, and adjustments are made. This workflow ensures a comprehensive and iterative approach to developing and selecting the most effective fraud detection model.

#### Theoretical justification for proposed model architecture

The proposed architecture integrates individual models, ensemble methods, and adaptive learning algorithms into a unified workflow designed for effective subscription fraud detection. The model development process involves preprocessing the dataset, constructing various machine learning models, tuning hyperparameters, evaluating performance on validation and test sets, and iteratively refining the system until satisfactory results are achieved.

**Fig. 1:**
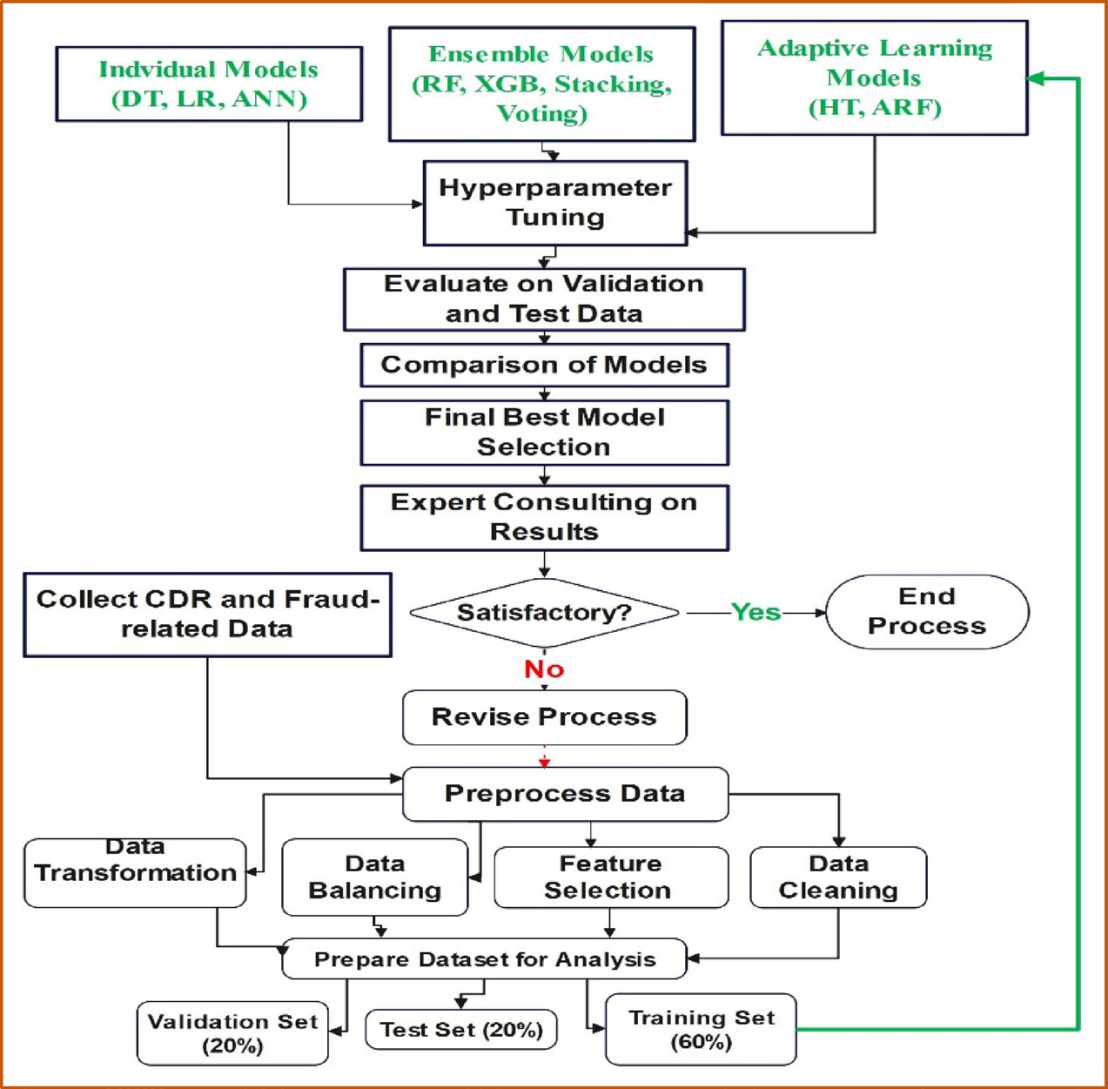
Proposed model architecture.

The overall workflow the above Fig. 1 begins by collecting CDR and fraud-related data, followed by essential preprocessing steps such as data cleaning, transformation, balancing, and feature selection. The prepared dataset is divided into training, validation, and testing subsets. Multiple classes of models—individual classifiers (DT, LR, ANN), ensemble methods (RF, XGBoost, Stacking, Voting), and adaptive learners (Hoeffding Tree, Adaptive Random Forest) are trained and compared. Hyperparameter tuning ensures that each algorithm operates at its optimal configuration. The final model is selected based on performance metrics and expert consultation.

The design of the proposed architecture is grounded in well-established theoretical principles in machine learning, ensemble learning, and data stream modeling. At the foundational level, the architecture employs diverse individual models, each representing a unique hypothesis function h_1_,h_2_,…,h_nh___1_, h__2_, …, h__nh1_​,h_2_​,…,h_n_​. According to supervised learning theory, diversity among these hypothesis functions increases the likelihood of capturing different aspects of the underlying decision boundary, thereby enhancing the model’s representational capacity.

The integration of ensemble methods is theoretically justified by ensemble learning research, which demonstrates that combining multiple learners yields a composite function H(x)H(x)H(x) with lower generalization error, provided the base models exhibit sufficient diversity^1^. Techniques such as Random Forest and XGBoost reduce variance and optimize loss functions through bagging and boosting, respectively. Stacking, in particular, is supported by meta-learning theory, where a secondary learner aggregates the outputs of base models to better estimate the conditional distribution P(y∣x)P(y|x)P(y∣x), effectively reducing correlated prediction errors^2^.

To address evolving fraud patterns, the architecture incorporates adaptive learning models**,** which are theoretically supported by data stream mining. Hoeffding Trees rely on the Hoeffding bound, providing a statistical guarantee that decisions made from small, incremental data samples converge toward optimal splits with high probability. Adaptive Random Forest extends this theory by integrating drift-detection mechanisms to maintain high performance under concept. These models ensure that the system remains robust in dynamic environments where fraud behavior continually changes^3^.

Finally, the iterative “evaluate → compare → revise” cycle in the workflow aligns with empirical risk minimization and model refinement principles. Hyperparameter tuning systematically explores the hypothesis space to optimize generalization performance, while expert consultation ensures alignment with domain knowledge and operational constraints.

#### Functioning and effectiveness of the proposed ensemble approach

The functioning of the proposed ensemble approach lies in its multi-stage integration of heterogeneous machine learning models, each contributing unique structural insights into the underlying fraud patterns. In the first stage, diverse base learners including Random Forest, Gradient Boosting, Logistic Regression, Artificial Neural Networks, and Adaptive Random Forest independently learn decision functions from the same data but capture different statistical regularities due to their distinct inductive biases and modeling assumptions. This diversity is critical, as ensemble theory has consistently shown that combining models with uncorrelated or weakly correlated errors leads to significant reductions in overall generalization error^4^. The second stage employs a stacking meta-learner, which is trained on out-of-fold predictions from the base models to prevent overfitting and to learn optimal model-specific weights. As demonstrated in modern stacking literature, meta-learners can approximate the Bayes-optimal combination of classifiers under mild regularity conditions, enabling improved calibration, adaptive weighting, and correction of systematic errors produced by individual models^5^. This operational workflow moving from diverse model generation to meta-level aggregation forms the core of the proposed architecture and ensures that each model’s strengths are effectively leveraged.

The effectiveness of this ensemble design is particularly pronounced in fraud detection, where severe class imbalance, dynamic fraud strategies, and feature heterogeneity require models that are both flexible and robust. Adaptive models such as the Adaptive Random Forest contribute resilience to evolving fraud patterns through incremental learning and drift detection, making the architecture suitable for non-stationary environments^6^. Simultaneously, boosting-based models reduce bias by iteratively correcting misclassified observations, while linear models improve probability calibration, strengthening overall predictive confidence. Combining these complementary capabilities increases both precision and recall, which is essential in domains where false negatives carry substantial financial risk. Empirical evidence from this study reinforces the theoretical expectations: the ensemble achieved higher PR-AUC, reduced false-negative rates, and improved F1-scores compared with all standalone models. These improvements align with contemporary findings that hybrid ensemble systems consistently outperform single classifiers in high-risk, imbalanced classification domains such as fraud detection^7^. Together, the workflow, theoretical justification, and performance outcomes demonstrate that the proposed ensemble approach provides a more accurate, stable, and generalizable framework for subscription fraud detection.

### Data collection and preparation

#### Data collection

Telecom companies typically manage millions of subscribers and maintain extensive databases to store information on these subscribers and their call records. Whenever a call is placed on a telecom network, detailed information about the call is stored as call detail record (CDR) data^11^. The dataset used for this research is sourced from Ethio Telecom.

- *Dataset Collection Sources*: The data was collected from various databases within the Ethio Telecom Information System Division, including the Customer Relationship Management (CRM) system and the Fraud Management System (FMS). The CRM system database contains customer information such as subscriber name, address, service plan, contract details, credit score, and payment history. The FMS database holds detailed records of fraud-related data^10^.
- *Dataset Collection Methods*: The CDR data was obtained from active mobile subscribers of Ethio Telecom, encompassing both prepaid and postpaid customers. The data collection process involved extracting Call Detail Records (CDRs) from Ethio Telecom’s databases over a specific two-month period known for increased fraudulent activity in the year 2023. In the prepaid service model, calls are made only when the subscriber makes a payment, making it easier for the service provider to manage and mitigate fraud. Prepaid services are generally less susceptible to fraud compared to postpaid services. In contrast, postpaid services involve billing the customer at the end of a billing cycle (commonly one to six months) based on usage, making it a more conventional service provided by telecom companies worldwide. The postpaid model, being a credit facility, presents a higher risk of fraud due to the deferred payment structure^10^.

#### Data description

To ensure ethical data use and protect customer privacy, all subscriber-level records used in this study were fully anonymized prior to analysis. Personally identifiable information—including names, phone numbers, device identifiers, addresses, and payment details was removed by the telecom operator using secure hashing and irreversible tokenization procedures. Sensitive attributes were either aggregated, masked, or transformed into non-identifiable numeric features, ensuring that no individual subscriber could be re-identified. The research team had access only to this anonymized dataset, which complied with the operator’s internal data governance policies and applicable data protection standards. No raw PII or operational identifiers were accessed or stored during the study. These privacy-preserving measures enhance transparency and ensure that the analysis was conducted in a secure, ethically responsible manner.

#### Explanation of raw data

The dataset utilized in this research comprises call detail records (CDRs) and fraud records from Ethio Telecom, spanning two months known for high fraud activity and including 1,000,000 records, covering both fraudulent and non-fraudulent cases. Each entry in the dataset includes multiple attributes that detail various elements of the call transactions and subscriber information. The dataset contains detailed telecommunications call records, where each entry is uniquely identified by a *Call_ID* and linked to a *Subscriber_ID*. It includes various features such as *Call_Type* (e.g., local, national), *Call_Duration*, *Call_Start_Time*, *Call_End_Time*, *Call_Cost*, and *Call_Status* (e.g., completed, dropped). It also records whether the call is *fraudulent* (*Fraud_Status*: Y/N), and tracks *Data_Usage_MB*, the number of *SMS_Sent* and *SMS_Received*, as well as *Subscription_Type* (e.g., prepaid, postpaid) and *Billing***_***Cycle*. Additional attributes include *Roaming***_***Status* to indicate if the subscriber was roaming during the call, and the *Gender* of the subscriber.

While not all attributes directly indicate fraudulent behavior, they collectively provide a comprehensive view of the subscriber’s activity. Attributes such as Call_Duration, Call_Cost, and Roaming_Status can reveal unusual patterns that may signal potential fraud. On the other hand, demographic information like Gender, while not directly related to fraud detection, offers additional context that can enhance the overall analysis. The Fraud_Status attribute serves as the critical target variable for training and evaluating the machine learning models, enabling the classification of calls as either fraudulent or non-fraudulent.

#### Data preparation

##### Initial data exploration and quality assessment

The dataset for this research comprises call detail records (CDRs) from Ethio Telecom, with a total of 1,000,000 records and 16 features, including both numerical (e.g., Call_duration, Call_Cost) and categorical (e.g., Subscription_ Type, Fraud_Status, Call_Status) data. Initial exploration involved generating descriptive statistics for numerical features, such as mean, median, and standard deviation, and frequency distributions for categorical features. Visual tools like box plots, were used to assess the distribution of numerical features and to identify any skewness. Additionally, scatter plots helped in visualizing relationships between key features.

During this exploration, we checked for data consistency, ensuring uniform date formats, consistent units of measurement, and logical consistency (e.g., ensuring that call end times were after call start times). Initial observations revealed a few anomalies, such as unusual spikes in call durations.

##### Handling missing values, outliers, and inconsistencies

*Missing Values*: Handling missing values is critical to avoid biased results and reduced statistical power in the study. Missing data was identified in features like call duration and call status. To address this, imputation techniques were used: the median value was employed for numerical features, as it is robust against outliers and provides a reliable central value, while categorical data was imputed using the mode, reflecting the most common patterns in the dataset^29^. This approach ensured that the dataset remained complete and representative, minimizing the impact of missing data on the model’s performance.

*Outliers*: Outliers, which can significantly skew results and affect model performance, were identified using Z-scores for numerical features and visualized with box plots. The Z-score method was particularly effective in subscription fraud detection, as it standardizes data and highlights anomalies by quantifying deviations from the mean. To manage the impact of these extreme values, a log transformation was applied to call duration, reducing the influence of outliers on the predictive model. This step was crucial in ensuring that the model accurately captured typical patterns without being distorted by extreme values^30^.

*Inconsistencies*: Inconsistencies in the data, such as duplicate records and varied date formats, were identified and addressed to maintain dataset reliability. Dates were standardized to a uniform format (YYYY-MM-DD), and categorical labels were converted to binary values (1 and 0) to ensure consistency across the dataset. Deduplication processes were also implemented to remove redundant records, further enhancing the integrity of the dataset. This meticulous approach to managing inconsistencies ensured that the data used in building the fraud detection model was both accurate and consistent, leading to more reliable model outcomes.

#### Feature engineering and selection

In this section, we describe detail the processes of feature engineering, which are critical steps in preparing the dataset for building effective predictive models.

##### Feature selection using correlation matrix

To refine the dataset further and ensure that the features contribute meaningfully to the model, a correlation matrix was generated. This matrix helps in understanding the relationships between features and identifying any multicollinearity issues^31^.

Correlation analysis was selected over other feature selection methods for this fraud detection study due to its ability to effectively identify and handle multicollinearity, feature redundancy, and model performance issues^31^. Highly correlated features can lead to multicollinearity, where one feature can be linearly predicted from another with a substantial degree of accuracy. This can distort the significance of predictors and affect the stability of the model coefficients^4^. By identifying and addressing multicollinearity through correlation analysis, we ensure that the model’s predictors remain significant and stable. Additionally, features that are highly correlated with each other provide redundant information. Reducing redundancy by selecting or dropping features helps in simplifying the model without losing predictive power. Simplified models are not only easier to interpret but also less prone to overfitting. Furthermore, removing or combining highly correlated features can improve the model’s performance by ensuring that each feature contributes unique information, thereby enhancing the model’s generalizability and robustness. The process of generating and analyzing the correlation matrix uses the below three step procedures^31^.

*Generate and Visualize the Correlation Matrix*: The correlation matrix was computed and visualized using a heatmap, which highlighted both positive and negative correlations between features. This visualization facilitated the identification of strongly correlated feature pairs.

*Analyze the Correlation Matrix:* A threshold of 0.8 was set to identify highly correlated pairs of features. Features with correlation coefficients above this threshold were considered highly correlated, indicating potential redundancy.

*Select or Drop Features:* Based on the correlation matrix analysis, features that were highly correlated with each other were considered for removal to reduce multicollinearity. The selection of features to drop was guided by domain knowledge and the importance of each feature in the context of fraud detection.

##### Feature selection using online streaming feature selection (OSFS)

To improve real-time fraud detection, the Fast Online Streaming Feature Selection (Fast-OSFS) method was used, enabling the model to adaptively select relevant features from continuous data streams. Unlike traditional static methods, Fast-OSFS updates feature sets in real time, allowing the model to respond to evolving fraud patterns without frequent retraining. This dynamic approach enhances the model’s efficiency and accuracy in detecting new fraud behaviors within constantly changing telecommunication environments^32^.

##### Feature engineering: creating new features from raw data

Feature engineering involves creating new features that can help improve the performance of the machine learning models. For this study, we derived several new attributes from the raw call detail records (CDRs) to enhance the predictive power of our models. These derived attributes are designed to capture essential patterns and relationships within the data that may be indicative of subscription fraud.

- **RATIO_UNIQUE_TOTAL**: This feature represents the ratio of unique calls to the total number of calls made by a subscriber. It is calculated as follows in below:
  1 $$RATION\_UNIQUE\_TOTAL= \frac{Number of Unique Calls}{Total Calls}$$

This ratio compares the number of unique calls made to the total number of calls. A high ratio indicates that most calls are made to unique numbers, which is normal behavior. Conversely, a low ratio might indicate repeated calls to the same numbers, which can be a red flag for fraud (e.g., call forwarding scams, telemarketing frauds).

This helps to identify patterns where fraudsters may make many unique calls as opposed to repetitive calls made by genuine users.

- RATIO_INT_TOTAL: This feature represents the ratio of international calls to the total number of calls. It is calculated as shown in below:
  2 $${\mathrm{RATIO}}_{{\mathrm{INT}}_{\mathrm{TOTAL}}}=\frac{\text{Number of International Calls Total Calls}}{\text{Total Calls}}$$

A higher ratio of international calls could indicate fraudulent activity, as fraudsters often exploit international calling plans.

This ratio shows the proportion of international calls relative to the total number of calls. Since international calls are often more expensive, a high ratio can be suspicious, particularly if the user does not usually make such calls. This could indicate a compromised account being used for fraudulent international calling.

### Feature scaling and normalization

Feature scaling and normalization are crucial preprocessing steps that ensure all features contribute equally to the model, and the training process is stable and efficient. Given the range of values in different features, it is essential to standardize these features to a common scale^31^.

- **Standardization**:
- This technique transforms the data to have a mean of zero and a standard deviation of one. It is particularly useful when the data follows a Gaussian distribution. The formula for standardization is as shown in below^31^.
  3 $$Standardize Value=\frac{Original Value -- Mean}{Standard Deviation}$$

Standardization ensures that features with large values do not dominate those with smaller values, which is crucial for algorithms like logistic regression and support vector machines^33^.

For this study, standardization is considered. Ultimately, standardization was chosen due to its effectiveness in handling features with different scales and distributions, especially given the various ratios and interaction terms created during feature engineering^34^.

The following steps were implemented for feature scaling:

1. Calculate the mean and standard deviation for each feature in the training set.
2. Transform the training set using these parameters to ensure that the transformed data has a mean of zero and a standard deviation of one.
3. Apply the same transformation to the test set using the mean and standard deviation calculated from the training set to maintain consistency.

This approach ensures that the models are trained on standardized data, leading to improved performance and stability.

#### Data splitting and sampling

In this section, we outline the methods used for splitting the data into training and testing sets, as well as the rationale behind the chosen splitting techniques. Additionally, we discuss the application of the SMOTE (Synthetic Minority Over-sampling Technique) algorithm combined with Tomek links.

### Data splitting method

In the model evaluation process, the data is divided into training, validation, and testing sets to ensure an unbiased assessment of the model’s performance. The training set is used to build the model, the validation set for tuning hyperparameters, and the testing set for evaluating generalization on unseen data. In this study, a 60-20-20 split was applied allocating 60% of the data for training, and 20% each for validation and testing to balance effective learning, fine-tuning, and robust evaluation^35^.

### Handling imbalanced data

Tomek Links, on the other hand, identifies and remove pairs of instances from different classes that are each other’s nearest neighbors, thereby cleaning the boundaries between classes and reducing overlap [51]. This combination offers several advantages for fraud detection: it improves the representation of the minority class, clarifies the distinction between fraud and non-fraud cases by cleaning class boundaries, and reduces the model’s bias towards the majority class, ultimately enhancing the model’s ability to detect fraudulent activities [53].

Tomek Links improve class separation by eliminating overlapping instance pairs from different classes that are nearest neighbors, thereby refining class boundaries. When integrated with SMOTE in the SMOTETomek method, this hybrid approach not only enhances the representation of the minority class but also reduces model bias toward the majority class, leading to better discrimination between fraudulent and non-fraudulent cases and improved fraud detection performance^36^.

What specific advantage of the SMOTE**–**Tomek hybrid method** (**SMOTETomek**)** enhances fraud detection by combining oversampling and data cleaning to address class imbalance. It improves the representation of fraudulent cases through synthetic oversampling (SMOTE) while refining class boundaries by removing overlapping instances using Tomek Links. This dual process reduces model bias toward non-fraudulent cases, resulting in clearer class separation and improved accuracy. Ultimately, SMOTETomek transforms an imbalanced dataset into a balanced one, enabling more effective discrimination between fraudulent and genuine transactions^36^.

### Model selection and development

The model selection and development process is a critical phase in this study, aimed at identifying and optimizing the most effective predictive models for subscription fraud detection. A variety of models were selected for evaluation, including individual models ensemble methods, and adaptive learning models, each chosen for its unique strengths and applicability to the problem at hand.

#### Individual models

The study first explores individual machine learning models such as Decision Tree (DT), Artificial Neural Network (ANN), and Logistic Regression (LR). These models were selected as they provide a solid baseline for comparison. Decision Trees are chosen for their simplicity and interpretability, making it easy to understand the decision-making process in fraud detection^37^. Artificial Neural Networks were included due to their ability to model complex relationships and interactions within the data, which is crucial in capturing intricate patterns associated with fraud^38^. Logistic Regression is selected for its effectiveness in binary classification problems and its ability to provide probabilities that are useful for threshold- based fraud detection^11^.

#### Ensemble models

To enhance predictive performance and robustness, ensemble learning techniques were selected, including Bagging (Random Forest), Boosting (XGBoost), Stacking, and Voting.

Random Forest (Bagging) was chosen for its ability to reduce overfitting by averaging multiple decision trees, which improves model stability and accuracy^16^. This method is particularly effective for handling large datasets with high dimensionality, as it can process numerous features while providing feature importance scores, which help in understanding the key factors contributing to fraud detection^39^.

XGBoost was selected within the Boosting category due to its strong performance in various machine learning tasks. XGBoost is known for its efficiency and speed, handling large-scale data effectively while preventing overfitting through regularization. Its ability to optimize both speed and accuracy makes it particularly well-suited for detecting complex fraud patterns in large datasets^39^.

Stacking was selected because it combines multiple base models into a single meta-model, leveraging the strengths of different algorithms to improve overall performance. In this study, the base models include a Decision Tree, Logistic Regression, and Artificial Neural Network^11^. These models were selected for their complementary strengths: Decision Trees providE clear decision rules, Logistic Regression offers interpretability and probability estimates, and Neural Networks capture complex patterns in the data. The meta-model integrates these diverse predictions, leading to a more accurate and robust fraud detection system.

Voting is another ensemble method included in the study, where multiple models specifically Decision Tree, Logistic Regression, and Artificial Neural Network are trained separately, and their predictions are combined through majority voting. This method is particularly useful in improving model robustness, as it leverages the strengths of these diverse algorithms and mitigates individual model weaknesses by aggregating their predictions^37^.

#### Adaptive learning models

Given the dynamic nature of fraud patterns, adaptive learning models were incorporated. Hoeffding Tree was chosen for its capability to handle streaming data incrementally, allowing the model to adapt in real-time as new data becomes available. This makes it particularly suitable for environments where fraud tactics evolve rapidly^40^.

Adaptive Random Forest (ARF) extends the traditional Random Forest to handle data streams, with mechanisms to detect and adapt to changes in data distribution. This adaptive capability is crucial for maintaining the relevance and accuracy of the fraud detection system over time^41^.

### Model development with hyperparameter tuning

Model development with hyperparameter tuning is essential for improving the accuracy and robustness of fraud detection systems. This involves selecting a base model, training it, and systematically adjusting its hyperparameters to optimize performance metrics such as accuracy, precision, recall, and F1-score^44^. Among the available tuning methods, grid search was chosen for this study due to its exhaustive evaluation of all possible hyperparameter combinations, enabling precise optimization. This thorough approach is particularly important for enhancing the model’s effectiveness in identifying fraudulent activities in the telecommunications sector^42^

Meticulous hyperparameter tuning was crucial in this study to enhance the accuracy and reliability of fraud detection models. Key parameters were optimized across various algorithms to balance bias-variance trade-offs and prevent overfitting. For Random Forest, parameters such as *n_estimators*, *max_depth*, and *min_samples_split* were adjusted; in XGBoost, tuning focused on *n_estimators*, *eta*, *max_depth*, and regularization terms (*alpha* and *lambda*); while Adaptive Random Forest involved optimizing *n_estimators*, *max_features*, and *max_depth* to improve adaptability in data streams. This systematic optimization process significantly improved performance metrics including accuracy, precision, recall, and F1-score strengthening the fraud detection system’s overall effectiveness^42^.

### Model evaluation

#### Evaluation metrics

Evaluating model performance is a critical step in ensuring the reliability and effectiveness of fraud detection systems, particularly in the presence of class imbalance. This study employs several key evaluation metrics: *Accuracy*, which measures the overall correctness of predictions but may be misleading in imbalanced datasets; *Precision*, which evaluates the proportion of true fraud detections among all predicted fraud cases and is vital for minimizing false positives; *Recall*, which measures the model’s ability to identify actual fraud cases, helping reduce the risk of missed fraudulent activities; and the *F1 Score*, which balances precision and recall, making it especially useful in imbalanced classification scenarios. By using this combination of metrics, the evaluation provides a comprehensive understanding of model performance, ensuring the selected models are well-optimized for detecting subscription fraud while minimizing both false alarms and missed detections^43^.

### Environment setup and tools

To implement the subscription fraud detection system, Python was chosen as the primary programming language due to its simplicity, extensive libraries, and robust community support, making it highly suitable for machine learning and data science tasks. Core libraries used included scikit-learn for standard ML algorithms, XGBoost for gradient boosting, River and scikit-multiflow for online and stream-based learning, and pandas and NumPy for data manipulation and computation. Visualization was handled using matplotlib and seaborn^37^. The computing environment was carefully configured with hardware comprising an Intel Core i7 or higher, 16 GB RAM, 500 GB SSD, and an optional NVIDIA GPU for accelerated training. Software setup included Windows 10 Pro × 64, Python via Anaconda Distribution 3 for efficient package management, and Jupyter Notebook 7.0.8 as the IDE for interactive development and visualization.

## Result and discussion

### Dataset preparation

**Fig. 2:**
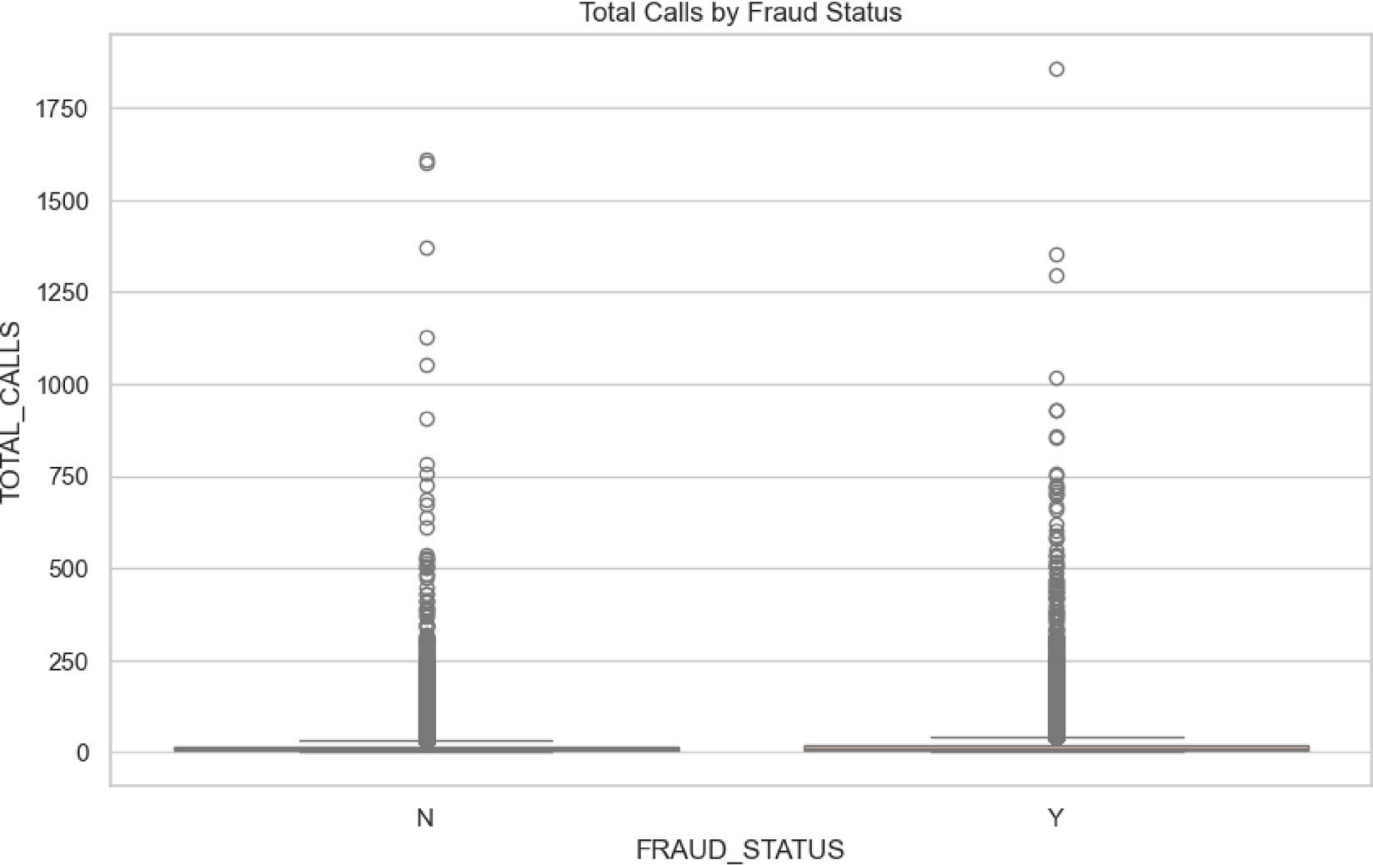
Total calls by fraud status.

*Exploratory Data Analysis*: Exploratory Data Analysis (EDA) provides essential insights into the dataset, focusing on key metrics relevant to subscription fraud detection^35^. This analysis examines total calls by fraud status, the ratio of unique to total calls, dialed call duration, data usage, and the ratio of international to total calls by fraud status. These metrics help uncover patterns and behaviors indicative of fraud, guiding the development of effective detection models. The Fig. 2 in below shows that the total Calls by Fraud Status.

The box plot compares total call distributions between non-fraud (N) and fraud (Y) cases, revealing that both categories have low medians and interquartile ranges, indicating limited variability and generally low call volumes. However, notable outliers exist in both groups, especially in fraud cases where they are slightly more frequent and higher, suggesting some fraudulent instances involve unusually high call activity. The overall symmetry in distributions, along with these outliers, points to the need for deeper analysis to uncover the factors behind such anomalies.

### Ratio of unique calls to total calls by fraud status

**Fig. 3:**
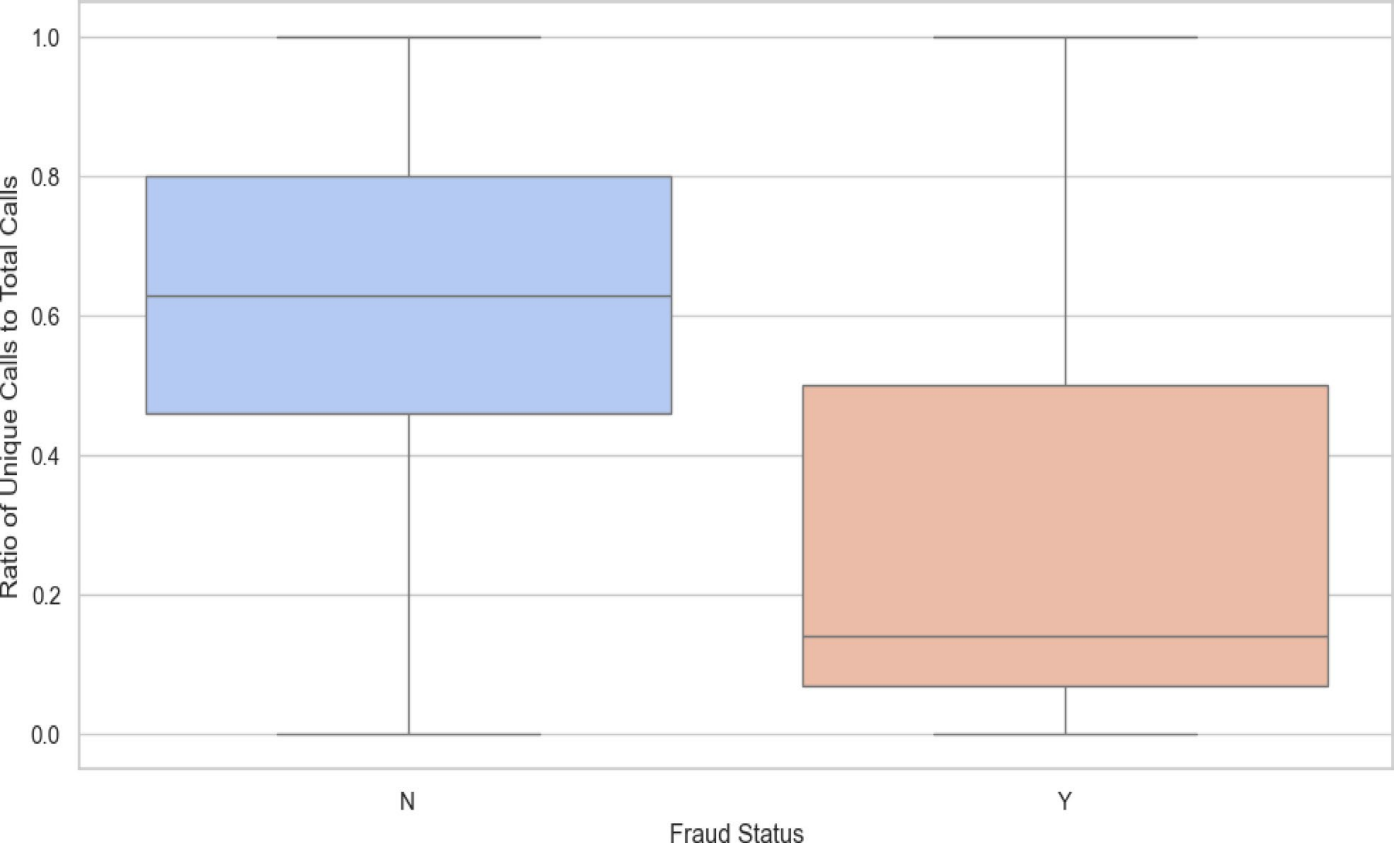
Ratio of unique calls to total calls by fraud status.

The Fig. 3 in the above illustarsts that, the analysis of the distribution of unique calls by fraud status reveals key insights. Both non- fraud (0) and fraud (1) cases show low medians and narrow interquartile ranges (IQRs), indicating that most instances involve a low number of unique calls with little variability. However, the median is slightly higher in fraud cases compared to non-fraud cases. The whiskers extend to similar ranges for both categories, suggesting comparable distributions of unique calls overall. Notably, both categories exhibit significant outliers, but the non-fraud category has outliers extending to much higher numbers of unique calls, indicating that while non-fraud cases generally involve fewer unique calls, some instances are exceptionally high. In contrast, fraud cases, although having some outliers, do not reach the same extremes. This suggests that unique calling patterns are more variable in non-fraud cases, while fraud cases are more consistent, with fewer extreme outliers.

**Fig. 4:**
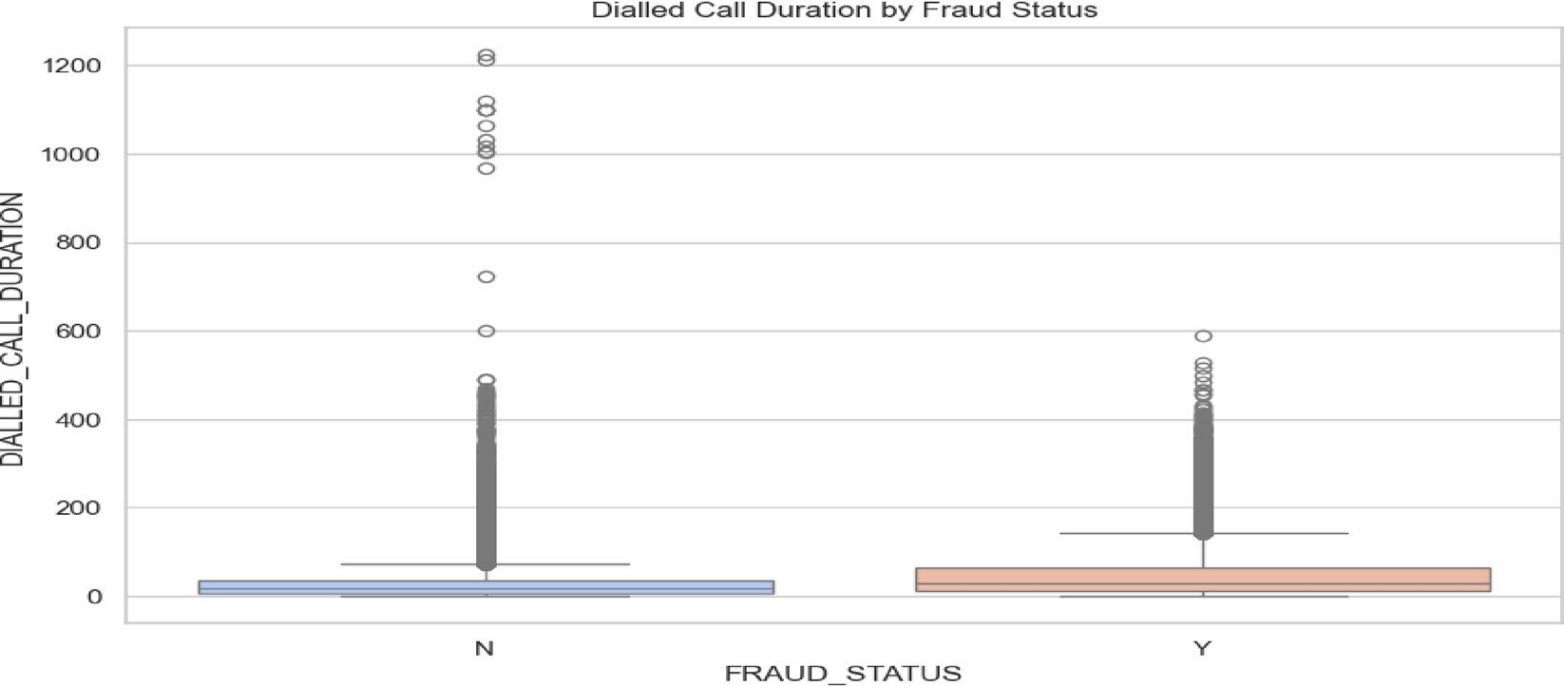
Dialed call duration by fraud status.

*Dialed Call Duration by Fraud Status*: This analysis of dialed call duration by fraud status reveals key distribution insights. For non- fraud (N) cases, the median call duration is relatively low, indicating that most calls are short, with a narrow interquartile range (IQR) reflecting low variability. In contrast, fraud (Y) cases also have a low median, but it is slightly higher than in non-fraud cases, suggesting marginally longer call durations on average. The IQR for fraud cases is broader, indicating greater variability in call durations. Both categories exhibit significant outliers, though non-fraud cases have outliers extending to much higher durations, indicating some exceptionally long calls. Fraud cases, while also showing outliers, do not reach the same extremes. As the Fig. 4 in the below, suggests that call duration patterns are more variable in non-fraud cases, whereas fraud cases are more consistent with fewer extreme outliers.

**Fig. 5:**
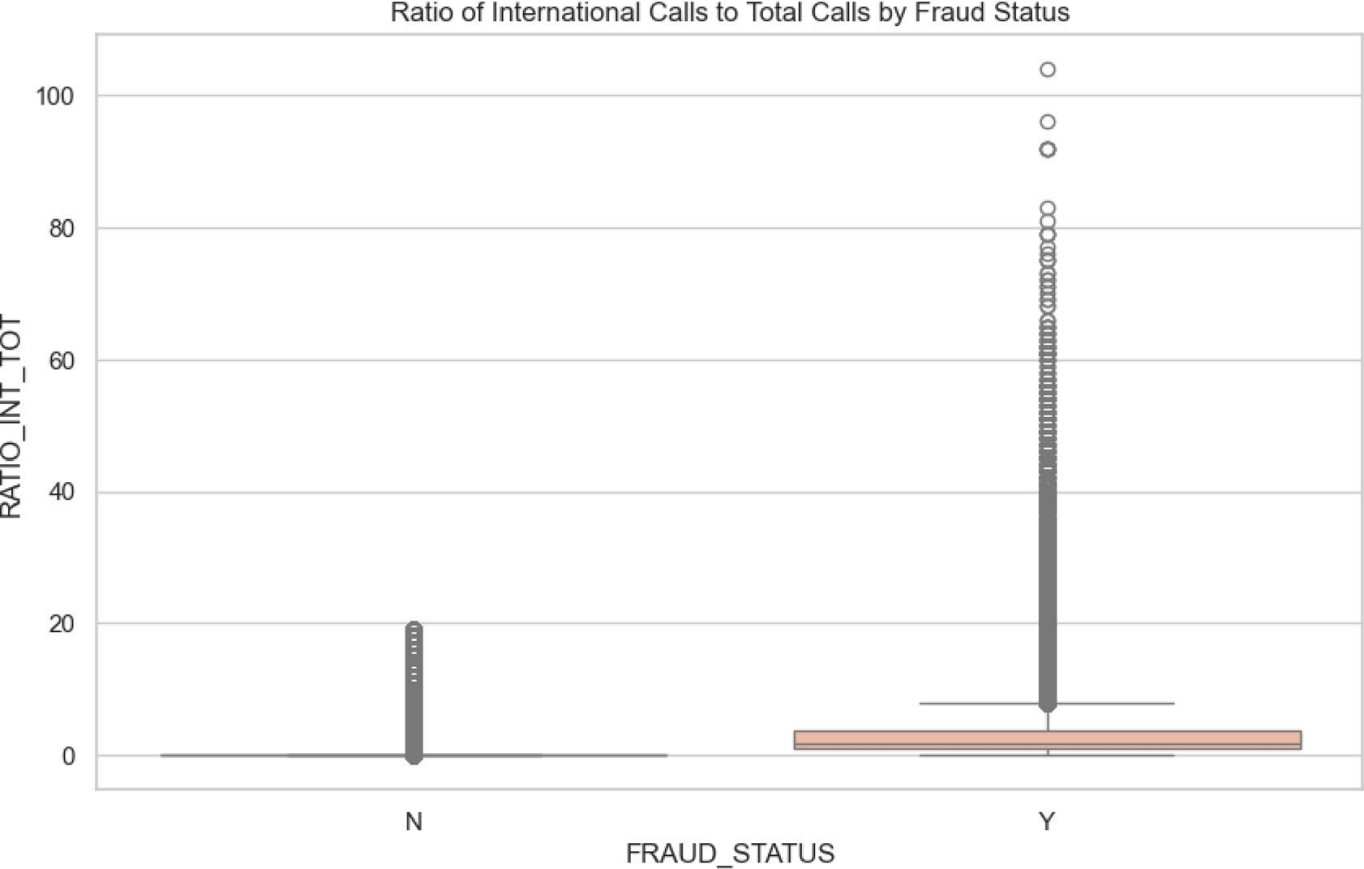
Ratio of international calls to total calls by fraud status.

*Ratio of International Calls to Total Calls by Fraud Status:* The Fig. 5 in belwo illustartes the analysis of the ratio of international calls to total calls by fraud status reveals distinct distribution patterns. For non-fraud (0) cases, the median ratio is very low, with an extremely narrow interquartile range (IQR), indicating that most non-fraud cases have a low proportion of international calls and little variability. In contrast, fraud (1) cases have a higher median ratio, suggesting that fraud cases tend to involve a greater proportion of international calls. The IQR for fraud cases is broader, reflecting more variability. The whiskers in the non-fraud category are short, showing that most data points are near the median, while the fraud category exhibits longer whiskers, indicating wider variability. Outliers are more prevalent and extreme in fraud cases, with some ratios significantly higher, suggesting that certain fraud cases involve a disproportionately high number of international calls. The symmetry in non-fraud cases contrasts with the broader distribution and more frequent outliers in fraud cases, indicating that fraudulent activities often involve a higher proportion of international calls. This suggests that monitoring the ratio of international calls could serve as a useful indicator for identifying potentially fraudulent activities.

**Fig. 6:**
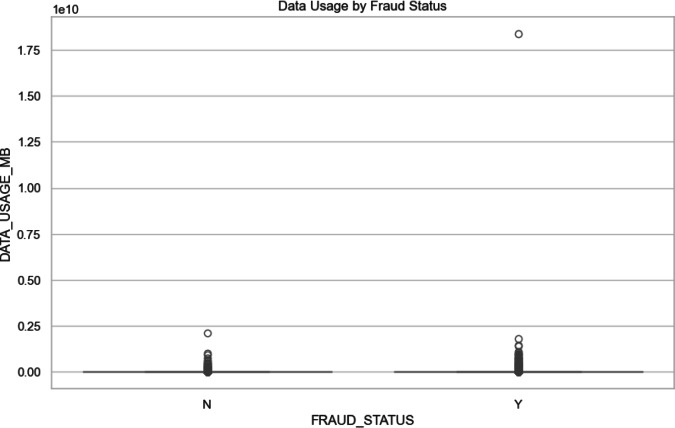
Data usage by fraud status.

*Data Usage by Fraud Status*: The Fig. 6 in blow shows, the analysis of data usage by fraud status provides key distribution insights. Both non-fraud (0) and fraud (1) cases exhibit very low median data usage, with narrow interquartile ranges (IQRs) indicating minimal variability in data usage across most cases. The whiskers are short for both categories, suggesting that most data points are clustered close to these low median values. However, outliers are present in both categories, with the non-fraud category showing a few cases of slightly higher data usage. Notably, the fraud category includes an extreme outlier with significantly higher data usage, suggesting that some fraudulent activities may involve exceptionally high data consumption. The distribution is generally symmetric for both fraud and non-fraud cases, but the presence of such extreme outliers in the fraud category highlights that certain fraud schemes may result in unusually high data usage, distinguishing these cases from typical patterns.

### Handling imbalanced data

**Fig. 7:**
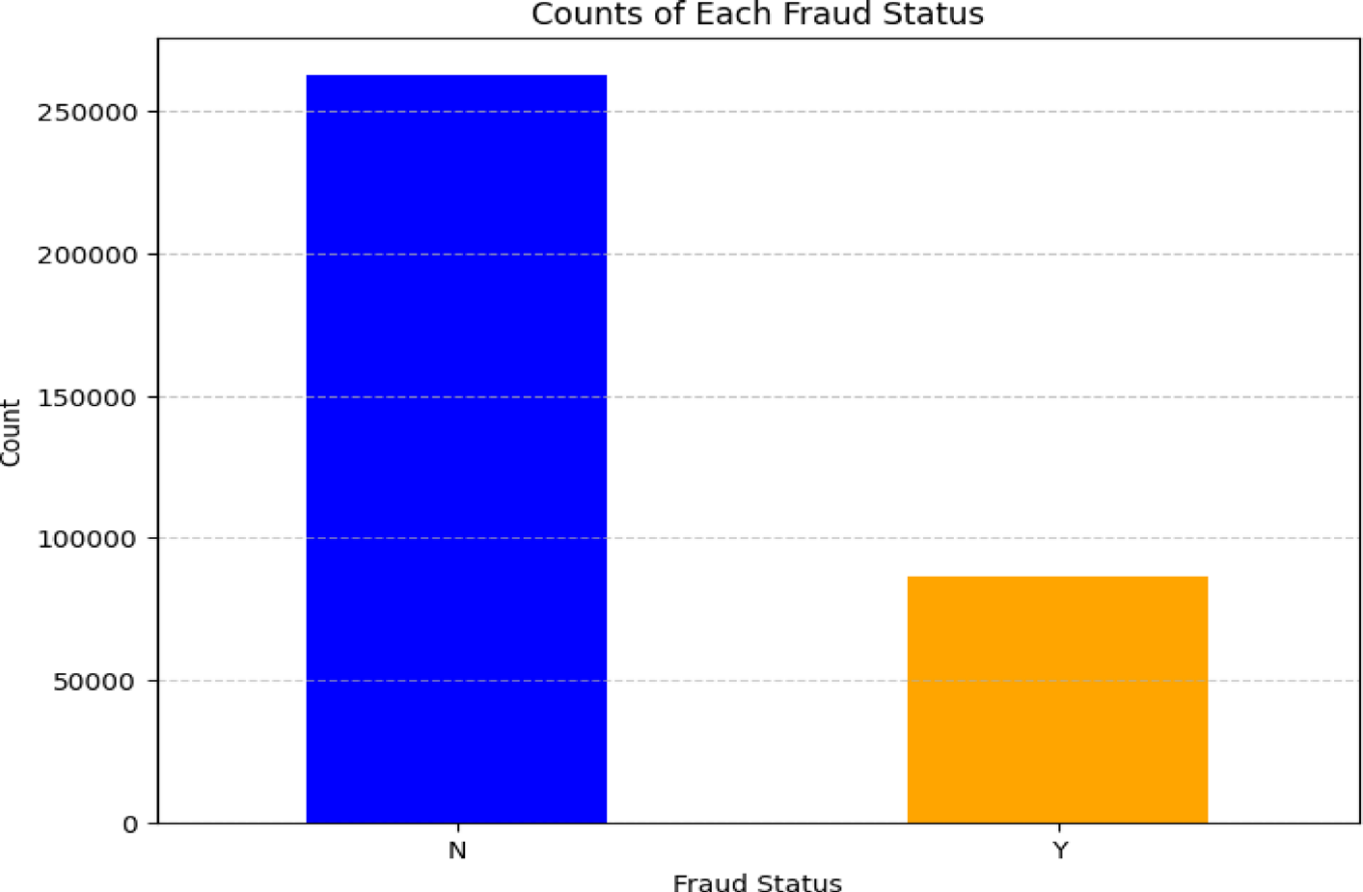
Original class distribution.

In this research, the dataset used is imbalanced, which poses a challenge for accurate fraud detection. To address this, SMOTETomek have been applied to ensure the model can effectively identify fraudulent cases. The Fig. 7 in below, present visualizations of the data both before and after balancing, illustrating how these techniques have adjusted the class distributions to improve model performance.

The above Fig. 7, illustrates the original class distribution of the dataset, highlighting the imbalance between non-fraudulent (N) and fraudulent (Y) cases. As depicted, there is a significantly higher number of non-fraudulent cases compared to fraudulent ones. This imbalance can lead to biased model predictions, making it crucial to apply balancing techniques to ensure accurate and fair fraud detection.

**Fig. 8:**
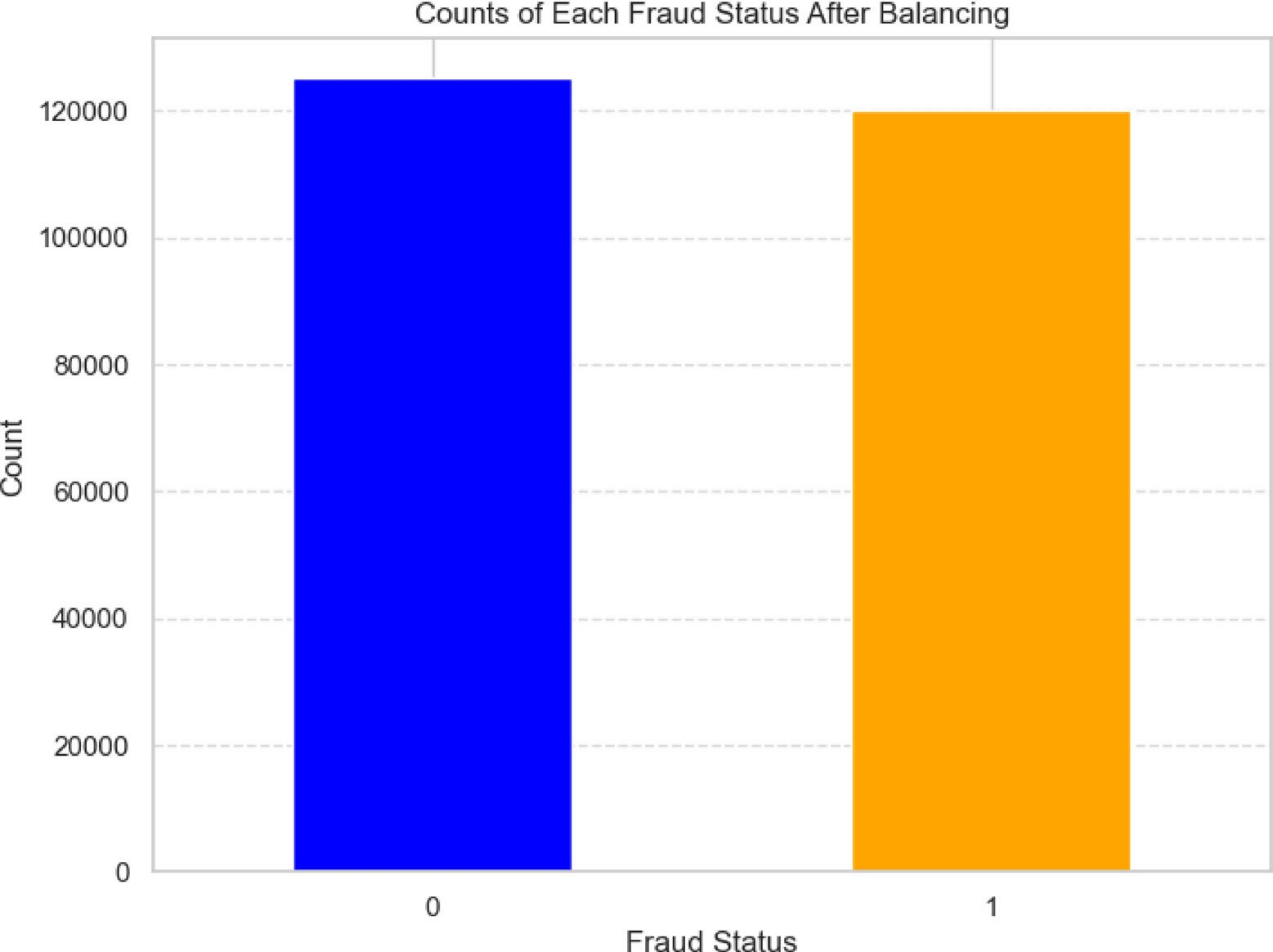
Class distribution after resampling.

The above Fig. 8 demonstrates the class distribution after applying the SMOTETomek resampling technique. The previously imbalanced dataset has now been balanced, with nearly equal counts of non-fraudulent (0) and fraudulent (1) cases. This balanced distribution is essential for enhancing the model’s ability to detect fraudulent activities with greater accuracy and reducing the bias toward the majority class.

### Feature selection

Feature selection is vital for optimizing subscription fraud detection models by identifying the most relevant features that boost predictive accuracy and reduce misclassifications. Random Forest (RF) ranks features based on their contribution to impurity reduction across decision trees, enabling the selection of the most influential variables. Confusion matrix analysis further supports this process by revealing the accuracy and error distribution of the model, guiding feature refinement. For real-time detection, the Fast Online Feature Selection (Fast-OSFS) method dynamically evaluates feature relevance and redundancy using mutual information, ensuring that only the most critical features are retained. Together, these techniques enhance the accuracy, efficiency, and adaptability of fraud detection systems^37^.

**Fig. 9:**
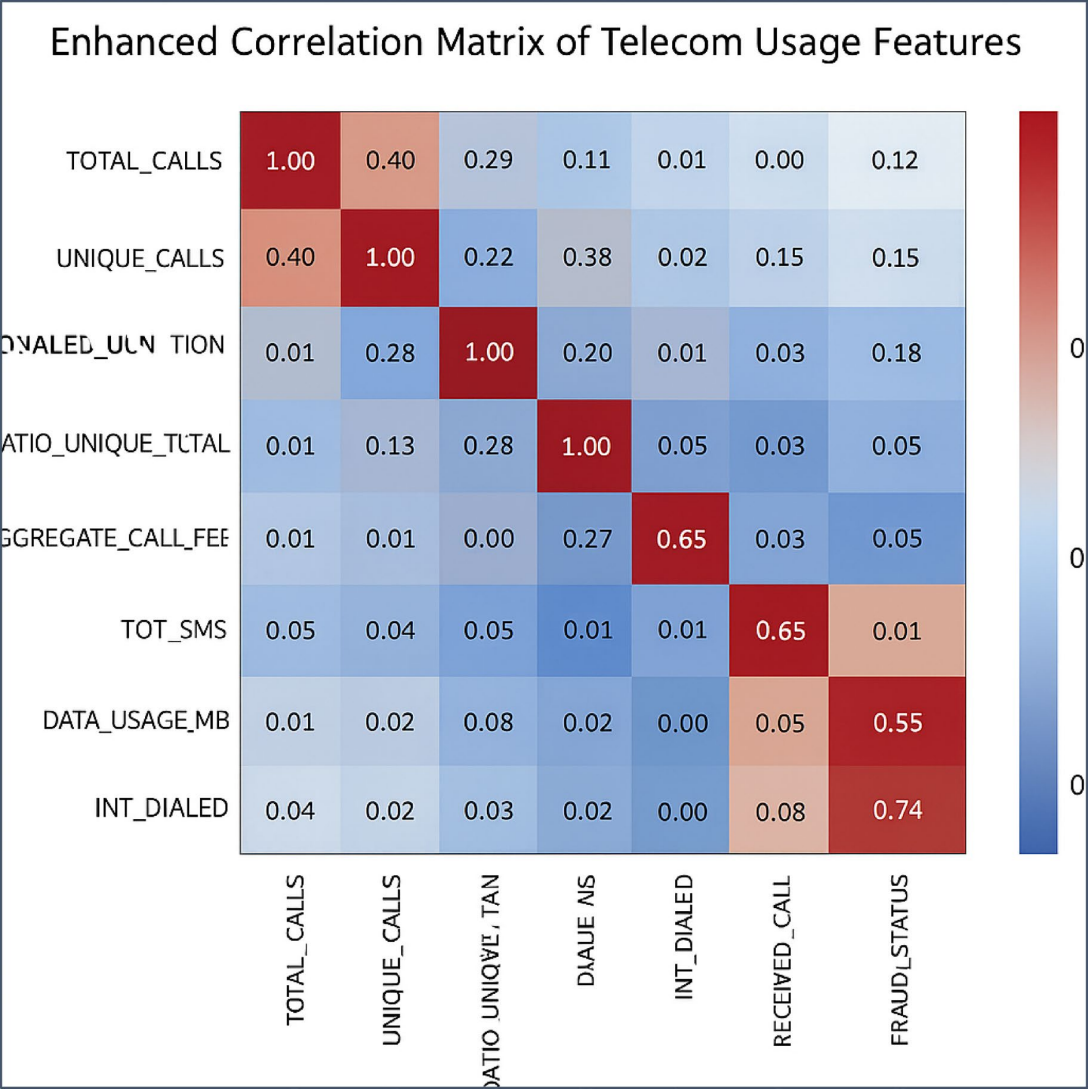
Correlation matrix analysis.

*Correlation Matrix Analysis: The above * Fig. 9* illustarts that, t*he correlation matrix analysis provided key insights into feature relationships relevant to fraud detection. A strong negative correlation between RATIO_UNIQUE_TOTAL and FRAUD_STATUS indicated that fraud often involves repeated calls, while positive correlations between INT_DIALLED and RATIO_INT_TOT with FRAUD_STATUS highlighted international call activity as a key fraud indicator. Conversely, TOTAL_CALLS and UNIQUE_CALLS showed a moderate positive correlation, reflecting typical, non-fraudulent behavior. Based on these correlations, features like AGGREGATE_CALL_FEE, DATA_USAGE_MB, and TOT_SMS, which showed weak relevance to FRAUD_STATUS, were removed or deprioritized. This selective approach refined the feature set, improving the model’s accuracy and efficiency in detecting subscription fraud.

#### RF Feature selection

**Fig. 10:**
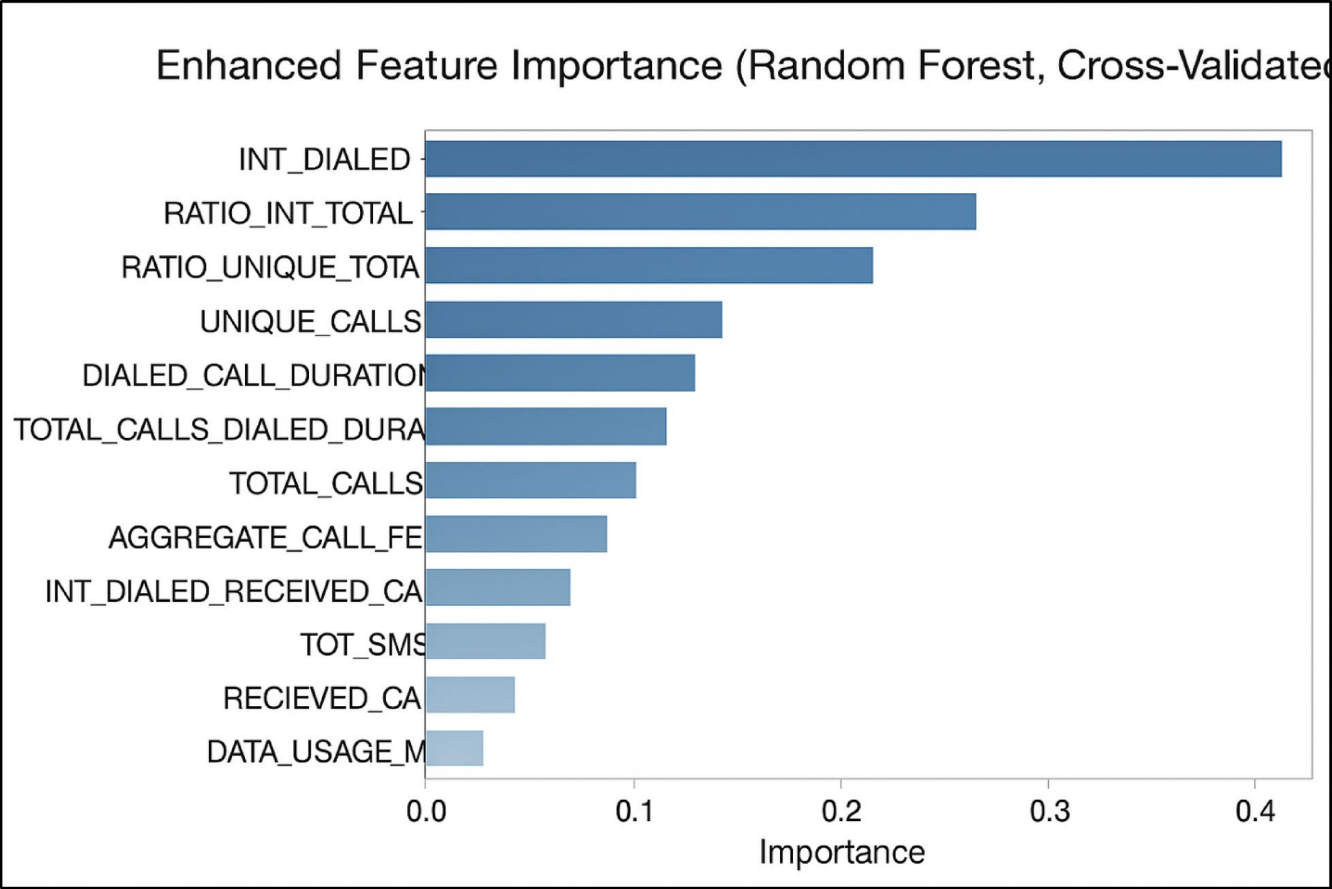
RF feature selection.

As indicated in the Fig. 10 above, the Random Forest feature importance analysis identified international calling behavior as the strongest indicator of subscription fraud, with *INT_DIALLED* emerging as the top predictor, followed by *RATIO_INT_TOTAL*, emphasizing the relevance of frequent international calls. *RATIO_UNIQUE_TOTAL* and *UNIQUE_CALLS* also proved important, suggesting that fraudsters often make repetitive calls to fewer numbers. Moderate contributors included *DIALLED_CALL_DURATION* and *TOTAL_CALLS_DIALLED_DURATION*, while features like *TOTAL_CALLS*, *AGGREGATE_CALL_FEE*, and *INT_DIALLED_RECEIVED_CALL* offered limited additional value. In contrast, *TOT_SMS****,**** RECEIVED_CALL*, and *DATA_USAGE_MB* had minimal impact and were excluded. The final model retained eight key features—those most correlated with fraud status and deemed most informative by the Random Forest analysis—enhancing the model’s accuracy and efficiency in detecting telecom fraud.

#### Online sequential feature selection (OSFS)

The Fast Online Sequential Feature Selection (Fast-OSFS) technique was used to enhance adaptive learning in fraud detection by dynamically selecting the most relevant features in real-time, allowing the model to efficiently adapt to shifting data patterns. While it operates like a “black box” and lacks the interpretability of methods like Random Forest or Correlation Matrix analysis, Fast-OSFS improves model performance by continuously focusing on the most predictive features and discarding irrelevant data. In subscription fraud detection, it proves particularly effective in tracking evolving indicators such as calling patterns and international communications, enabling timely responses to emerging fraud strategies^44^.

### Model building experiment using individual models

#### Decision tree results

The Table 2 in below, *Summary of Decision Tree performance*, presents a comparative analysis of a decision tree’s effectiveness before and after a tuning process. It evaluates the model’s capabilities using several key metrics: *accuracy, precision, recall, and F1 score*. For each metric, the performance is reported for both a *validation set and a test set,* offering insights into how well the model generalizes to unseen data. Notably, the accuracy, recall, and F1 score generally show improvement after tuning, particularly on the test set, while *precision slightly decreases on the test set post-tuning.* This indicates that the adjustments made to the decision tree aimed at optimizing its overall predictive power and generalizability.

**Table 2:** Summary of decision tree performance.

Metric	Before Tuning		After Tuning	
	Validation Set	Test Set	Validation Set	Test Set
Accuracy	0.9914	0.9889	0.9928	0.9903
Precision	0.9918	0.9749	0.9903	0.9681
Recall	0.9906	0.9805	0.9950	0.9937
F1 Score	0.9912	0.9777	0.9926	0.9807

As shown in the table above, the Decision Tree (DT) model demonstrates strong and consistent performance in detecting subscription fraud, achieving high accuracy (0.9844 on the validation set and 0.9831 on the test set), along with impressive precision, recall, and F1 scores across both datasets. With precision scores of 0.9862 and 0.9581, and recall values of 0.9819 and 0.9745 for validation and test sets respectively, the model effectively identifies and captures fraudulent cases. The F1 Scores of 0.984 (validation) and 0.9662 (test) further highlight its robust and reliable classification capabilities.

#### Logistic regression results

As summerized in Table 3 below:

**Table 3:** Summary of Logistic Regression performance.

Metric	Before Tuning		After Tuning	
	Validation Set	Test Set	Validation Set	Test Set
Accuracy	0.8284	0.7462	0.9574	0.9437
Precision	0.7443	0.4937	0.9361	0.8278
Recall	0.9888	0.9880	0.9799	0.9755
F1 Score	0.8493	0.6584	0.9575	0.8956

As indicated in the Table 3 abvoe, using the best hyperparameters {'C': 1, ‘penalty’: ‘l1’, ‘solver’: ‘liblinear’}, the Logistic Regression model demonstrates good performance with accuracy scores of 0.957 on the validation set and 0.944 on the test set. It achieves high recall (97.99% validation, 97.55% test), effectively identifying true positives, but its lower precision (0.936 validation, 0.828 test) indicates a higher rate of false positives. The resulting F1 Scores of 0.958 (validation) and 0.896 (test) show a solid overall performance, though improvements in precision would enhance the model’s reliability.

#### Artificial neural network (ANN) results

The Table 4 in below summerises the experimentation results of Artificial Neural Network Algorithm.

**Table 4:** Summary of artificial neural network performance.

Metric	Before Tuning		After Tuning	
	Validation Set	Test Set	Validation Set	Test Set
Accuracy	0.9671	0.9579	0.9676	0.9545
Precision	0.9532	0.8709	0.9472	0.8541
Recall	0.9809	0.9743	0.9888	0.9845
F1 Score	0.966	0.9197	0.9676	0.9147

As shown in the Table 4 above, the Artificial Neural Network (ANN) model, optimized with parameters {‘hidden_layer_sizes’: (100, 50), ‘max_iter’: 200, ‘solver’: ‘adam’}, demonstrates strong performance with high accuracy (0.968 validation, 0.955 test) and recall (98.88% validation, 98.45% test), effectively identifying true fraud cases. However, its lower precision (94.72% validation, 85.41% test) leads to a higher rate of false positives, impacting its F1 Scores (0.968 validation, 0.915 test). While the ANN shows impressive results, the Decision Tree model outperforms it overall by achieving more balanced and consistently high metrics across both datasets, making it the most reliable single model for subscription fraud detection among those evaluated.

### Model building experiment using ensemble models

#### XGBOOST algorithm

The results of the XGBoost algorithm, both before and after hyperparameter tuning, are discussed below, along with the results summary in the Table 5.

**Table 5:** Summary of XGBoost model performance.

Metric	Before Tuning		After Tuning	
	Validation Set	Test Set	Validation Set	Test Set
Accuracy	0.9946	0.9916	0.9985	0.9916
Precision	0.9933	0.9736	0.9981	0.9771
Recall	0.9956	0.9931	0.9988	0.9893
F1 Score	0.9944	0.9832	0.9985	0.9832

As the Table 5 in the above shows that, the XGBoost model, optimized with parameters such as a learning rate of 0.1, 200 estimators, and max depth of 5, demonstrates excellent performance in subscription fraud detection. It achieves high accuracy (0.9946 validation, 0.9917 test), near-perfect recall (0.9956 validation, 0.9931 test), and strong F1 scores (0.9946 validation, 0.9833 test), indicating its effectiveness in identifying fraudulent cases. Although there is a slight drop in precision on the test set (0.9737), the model maintains a strong balance between precision and recall, supported by a high ROC AUC of 0.9946. These results confirm XGBoost’s reliability and strong generalization capabilities, making it a top-performing model for accurately detecting subscription fraud.

#### RF algorithm

The Table 6 in below shows that, the results of the RF algorithm, both before and after hyperparameter tuning, are discussed below, along with the results summary.

**Table 6:** Summary of RF performance.

Metric	Before Tuning		After Tuning	
	Validation Set	Test Set	Validation Set	Test Set
Accuracy	0.9944	0.9915	0.9985	0.9916
Precision	0.9937	0.9786	0.9982	0.9771
Recall	0.9949	0.9786	0.9988	0.9894
F1 Score	0.9943	0.9830	0.9985	0.9832

The Bagging Random Forest (RF) model, optimized with parameters {‘max_features’: 1.0, ‘max_samples’: 1.0, ‘n_estimators’: 200}, demonstrates outstanding performance in detecting subscription fraud, achieving near-perfect metrics on the validation set with 0.9985 accuracy, 0.9982 precision, and 0.9988 recall, along with a ROC AUC of 0.9986. On the test set, the model continues to perform strongly with 0.9916 accuracy, 0.9771 precision, 0.9894 recall, and a F1 score of 0.9832, indicating excellent generalization and balance between false positives and false negatives. These results highlight the RF model’s robustness and effectiveness in real-world telecom fraud detection scenarios, making it a highly reliable tool for accurate and consistent identification of fraudulent activities.

#### Stacking method

The Table 7 in below shows that, the results of the Stacking algorithm, both before and after hyperparameter tuning, are discussed below, along with the results summary.

**Table 7:** Summary of stacking method performance.

Metric	Before Tuning		After Tuning	
	Validation Set	Test Set	Validation Set	Test Set
Accuracy	0.9947	0.990	0.9985	0.9925
Precision	0.9941	0.976	0.9981	0.9832
Recall	0.9949	0.989	0.9988	0.9945
F1 Score	9945	0.982	0.9985	0.9895

The Table 7 in the above summerises, the Stacking method, with individually tuned base classifiers and meta-classifier, delivers impressive results in subscription fraud detection, achieving high accuracy scores of 0.9985 on the validation set and 0.9925 on the test set. The model demonstrates strong recall particularly on the test set (0.9945) indicating its effectiveness in identifying true positives, while maintaining solid precision. The consistently high F1 Score, especially 0.9895 on the test set, reflects a well-balanced performance between precision and recall. These results highlight the Stacking model’s robustness, strong generalization ability, and suitability for handling complex classification tasks like subscription fraud detection.

#### Voting method

The Table 8 in below shows that, the results of the Voting algorithm, both before and after hyperparameter tuning, are discussed below, along with the results summary.

**Table 8:** Summary of voting performance.

Metric	Before Tuning		After Tuning	
	Validation Set	Test Set	Validation Set	Test Set
Accuracy	0.9934	0.9905	0.9985	0.9916
Precision	0.9923	0.9730	0.9982	0.9771
Recall	0.9940	0.9915	0.9988	0.9894
F1 Score	0.9931	0.9820	0.9985	0.9832

As the Table 8 in above summerises, Voting algorithm, using tuned hyperparameters of its base classifiers, demonstrates exceptional performance in subscription fraud detection, achieving high accuracy scores of 0.9985 on the validation set and 0.9916 on the test set. It maintains strong precision and recall, with recall slightly higher on the test set (0.9894), indicating its effectiveness in correctly identifying fraudulent cases. The high F1 Score of 0.9832 on the test set reflects a balanced performance in minimizing both false positives and false negatives. These results underscore the Voting method’s robustness, reliability, and strong generalization capability, making it highly suitable for detecting subscription fraud.

### Model building experiment using adaptive models

#### Hoeffding tree

The Table 9 in below shows that, the results of the Hoeffding algorithm, both before and after OSFS, are discussed below, along with the results summary.

**Table 9:** Summary of Hoeffding tree performance.

Metric	Before OSFS		After OSFS	
	Validation Set	Test Set	Validation Set	Test Set
Accuracy	0.9868	0.9834	0.9874	0.9848
Precision	0.9840	0.9477	0.9833	0.9496
Recall	0.9889	0.9874	0.9910	0.9914
F1 Score	0.9865	0.9672	0.9871	0.9701

The Table 9 in the above summerises, the Hoeffding Tree model shows solid performance in detecting telecom subscription fraud, with high accuracy (0.9874) and balanced precision (0.983) and recall (0.99) on the validation set, resulting in an F1 score of 0.987. On the test set, its precision decreases to 0.946, indicating more false positives, but recall remains strong at 0.9914, maintaining effective fraud identification. Despite a lower F1 score of 0.9701 compared to other models, the Hoeffding Tree’s key strength lies in its adaptive learning capability, allowing it to update continuously with streaming data and adapt to evolving fraud patterns in real-time, making it a valuable tool for dynamic fraud detection in telecommunications.

#### Adaptive random forest

The Table 10 in belows shows that, the results of the Hoeffding algorithm, both before and after OSFS, are discussed below, along with the results summary.

**Table 10:** Summary of adaptive random forest performance.

Metric	Before OSFS		After OSFS	
	Validation Set	Test Set	Validation Set	Test Set
Accuracy	0.990	0.989	0.9933	0.992
Precision	0.975	0.972	0.980	0.977
Recall	0.988	0.987	0.993	0.991
F1 Score	0.983	0.980	0.988	0.984

As the Table 10 above shows that, the Adaptive Random Forest (ARF) model shows strong and balanced performance in telecom subscription fraud detection, achieving high accuracy (0.9933 validation, 0.992 test), precision (~ 0.98 validation, 0.977 test), and recall (~ 0.993 validation, 0.991 test), with F1 scores of 0.988 and 0.984, respectively. Unlike batch learning models, ARF’s online learning capability allows it to adapt continuously to evolving fraud patterns in real-time, making it highly effective in dynamic environments. As an ensemble method combining multiple decision trees with drift detection, ARF reduces variance and improves robustness and accuracy, outperforming single-tree models like the Hoeffding Tree in both adaptability and detection effectiveness.

### Evaluation and comparison of the algorithms

#### Comparative analysis of performance metrics across different models

This section compares the performance metrics of the models implemented using ensemble models, adaptive models and individual models. The comparison highlights the strengths and weaknesses of each approach in terms of accuracy, precision, recall, F1 score, and ROC AUC. The Table 11 in belows shoas thet. The comparison of ensemble, adaptive and individual models within Performance Metrics Table.

**Table 11:** Comparison of ensemble, adaptive and individual models.

Metric	Ensemble Learning Models				Individual Models			Adaptive Models	
	XGBoos t	Bagging	Stacking	Voting	DT	LREG	ANN	ARF	HT
Accuracy	0.992	0.992	0.9925	0.992	0.983	0.944	0.95 5	0.99 2	0.984 8
Precision	0.974	0.977	0.9832	0.977	0.958	0.828	0.85 4	0.97 7	0.949 6
Recall	0.993	0.989	0.9945	0.989	0.975	0.976	0.98 5	0.99 1	0.991 4
F1 Score	0.983	0.983	0.9895	0.983	0.96 6	0.896	0.91 5	0.98 4	0.970 1

The Table 11 in the above shows, the comparison of ensemble, adaptive, and individual models highlights the Stacking model and Adaptive Random Forest (ARF) as the top performers in subscription fraud detection for Ethio Telecom. Both models achieve superior accuracy, precision, recall, and F1 scores, effectively identifying fraudulent activities while minimizing false negatives critical for reducing undetected fraud and financial losses. The Stacking model excels by combining predictions from multiple base classifiers to optimize detection, whereas ARF’s real-time adaptability makes it highly effective in the dynamic telecom environment. Together, these models offer a robust, accurate, and adaptable solution that significantly enhances Ethio Telecom’s fraud detection capabilities and financial security.

#### Visualization of model performance

In subscription fraud detection, effective visualization of model performance is essential for understanding the strengths and weaknesses of different approaches. This research emphasizes evaluating and comparing individual, adaptive, and ensemble machine learning models to improve the accuracy and reliability of detecting fraudulent subscriptions. The visualizations aim to offer a clear and comprehensive comparison of these models based on key performance metrics.

#### ROC curve visualization

To compare the performance of individual, ensemble, and adaptive models in subscription fraud detection, ROC (Receiver Operating Characteristic) curves are used, plotting true positive rate against false positive rate across thresholds. The visualization includes ROC curves for three individual models (Decision Tree, ANN, Logistic Regression), four ensemble methods (Bagging-RF, Boosting-XGB, Stacking, Voting), and two adaptive methods (Hoeffding Tree and Adaptive Random Forest), providing a clear comparison of their classification effectiveness.

**Fig. 11:**
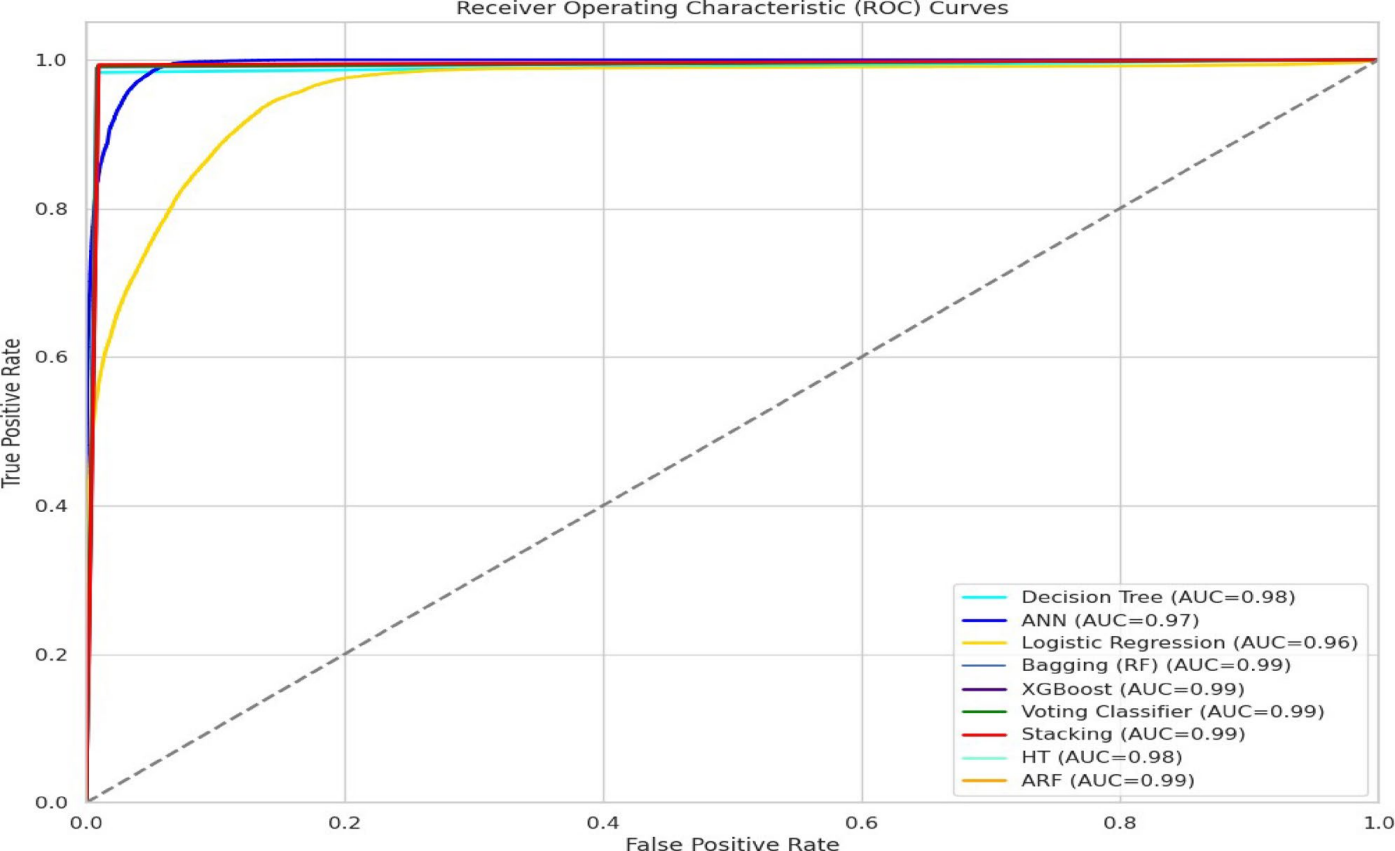
ROC curve visualization.

The Fig. 11 in above shows that, the ROC curve analysis shows that ensemble and adaptive models outperform individual models in subscription fraud detection, with ensemble methods achieving near-perfect AUC values (~ 0.99), indicating high effectiveness in distinguishing fraud from non-fraud cases. Among them, the Stacking model performs best due to its heterogeneous structure, combining diverse base models for greater predictive accuracy. Adaptive Random Forest (ARF) also stands out with an AUC of 0.99, leveraging its ability to adapt to evolving fraud patterns. Among individual models, Decision Tree leads with an AUC of 0.98, followed by ANN (0.97) and Logistic Regression (0.96). Overall, Stacking and ARF emerge as the most reliable models for accurate and adaptive fraud detection at Ethio Telecom.

### Discussion of the results with domain experts

#### Interpretation of the experimental results

The experimental results demonstrate that ensemble and adaptive learning models, particularly Stacking and Adaptive Random Forest (ARF), are highly effective in detecting subscription fraud, consistently outperforming individual models across metrics such as accuracy, precision, recall, F1 score, and ROC AUC. Their high accuracy ensures correct classification, while strong recall and precision help minimize false negatives and improve fraud detection rates. Discussions with domain experts validated these findings, highlighting the practicality of these models in real-world telecom environments. Experts emphasized the importance of adaptive learning for tracking evolving fraud patterns and confirmed the relevance of key features like international calling behavior. They recommended integrating these models into existing fraud detection systems to enhance predictive accuracy and maintain system effectiveness against emerging threats.

A closer examination of the confusion matrices provides deeper insight into the model’s misclassifications. False negatives instances where fraudulent activity is incorrectly classified as legitimate represent the most critical error type in telecom fraud detection, as they allow fraudsters to continue exploiting the system. The proposed ensemble significantly reduces false negatives compared with individual models, indicating improved sensitivity to subtle fraud patterns. Conversely, false positives legitimate subscribers incorrectly flagged as fraudulent can lead to unnecessary account verification steps or service interruptions. While the ensemble achieves a modest reduction in false positives relative to several baseline models, the trade-off between precision and recall remains evident. These misclassification patterns highlight the ensemble’s strength in minimizing undetected fraud while maintaining acceptable levels of false alarms. However, they also indicate that further refinement—particularly in calibrating decision thresholds and integrating cost-sensitive learning may help reduce remaining false positives without compromising fraud detection performance.

## Discussion of results

The results obtained in this study indicate that the proposed ensemble approach performs well within the evaluated dataset and experimental conditions, demonstrating improved precision–recall performance and reduction in false negatives compared with individual models. However, these findings should be interpreted as promising rather than conclusive. Because the evaluation was conducted using data from a single operational context, additional testing on external, independently sourced datasets is required to assess the model’s generalizability. Moreover, validation under real-world deployment conditions where fraud patterns evolve and operational constraints differ would provide a more comprehensive understanding of the model’s practical effectiveness. Thus, while the ensemble shows strong potential, its broader applicability remains subject to further empirical confirmation.

## Conclusions

This thesis thoroughly investigated the impact of ensemble and single-based classification methods for fraud detection, following a structured research methodology that included problem analysis, data collection and preprocessing, exploratory data analysis, model selection, training, evaluation, hyperparameter tuning, and deployment.

The study used a large Call Detail Record (CDR) dataset initially containing around 1,000,000 records, which was refined to 349,164 relevant records with eight key attributes. The data was thoroughly preprocessed for machine learning tasks, and all experiments were conducted using the Anaconda Jupyter Notebook platform.

The experimentation involved implementing single models (Decision Tree, ANN, Logistic Regression), ensemble models (Bagging, Boosting, Stacking, Voting), and adaptive models (Hoeffding Tree, Adaptive Random Forest) for subscription fraud detection. Model selection was guided by literature review, and performance was further improved through hyperparameter tuning to optimize key parameters.

Model performance was evaluated using standard metrics such as accuracy, recall, precision, F-measure, and ROC curve analysis. The Stacking model emerged as the most effective, achieving 99.28% accuracy and consistently outperforming both individual and other ensemble models by combining ANN, DT, and LR. Among adaptive models, Adaptive Random Forest outperformed the Hoeffding Tree, proving highly effective for evolving fraud detection strategies.

The experimental results were validated by domain experts, confirming the practical value of the developed models in subscription fraud detection. The study’s ensemble methods, particularly the Stacking model and Adaptive Random Forest, showed significant accuracy improvements over individual classifiers. These top-performing models offer strong potential for real-time fraud detection and adaptability to evolving fraud patterns, with hyperparameter tuning playing a key role in enhancing their effectiveness.

This study has several limitations that should be acknowledged. First, the evaluation was conducted using data from a single telecom environment, which may limit the generalizability of the results to other regions or operational contexts. Second, although the ensemble approach demonstrated strong performance on the available dataset, external validation using independent and more diverse datasets is needed to confirm its broader applicability. Third, the data reflect historical fraud behaviors, and the model may be sensitive to concept drift as fraud tactics evolve over time. While the inclusion of Adaptive Random Forest partially addresses this issue, further investigation into continual learning and real-time adaptation is required. Finally, the study did not assess constraints related to real-world deployment such as computational overhead, integration into existing fraud management systems, and latency requirements which may influence practical performance. These limitations provide useful directions for future research and highlight the importance of broader validation before large-scale deployment.

## Recommendations

Machine learning techniques are increasingly applied in telecommunications to support decision-making, particularly in fraud detection. This study demonstrated the effectiveness of classification and prediction methods for identifying subscription fraud in Ethio Telecom’s data. Based on the findings, several recommendations are proposed: Ethio Telecom should expand fraud detection efforts beyond subscription fraud to other types, using dynamic strategies; future research should incorporate network and time-series data; ensemble models like Stacking and Adaptive Random Forest (ARF) should be prioritized; integrating new features and external data can enhance accuracy; and tools like Apache Kafka are recommended for real-time monitoring of adaptive models. These insights aim to guide future research and strengthen fraud detection systems.

## Data Availability

The datasets generated and/or analyzed during the current study are available from repository at Kaggle: [https://www.kaggle.com/datasets/esubalewasmare/telecom-datasets](https:/www.kaggle.com/datasets/esubalewasmare/telecom-datasets).

## Abbreviations

Adaboost: Adaptive boosting
ANN: Artificial neural networks
ARF: Adaptive random forest
BSS: Business support subsystem
CDR: Call detail record
CFCA: Communications fraud control survey
CM: Confusion matrix
CRM: Customer relationship management
CSV: Comma separated values
DT: Decision tree
EDA: Exploratory data analysis
ETC: Ethiopian telecommunication corporation
FN: False negative
FMS: Fraud management systems
FP: False positive
Gb: Gradient boosting
GSM: Global system for mobile communication
HT: Hoeffding tree
IRSF: International revenue share fraud
KNN: K nearest neighbor
LR: Logistic regression
ML: Machine learning
MLP: Multilayer perceptron
OSFS: Online streaming feature selection
OSS: Operations support subsystem
PBX: Private branch exchange
PRF: Premium rate fraud
RA: Revenue assurance
RF: Random forest
ROC: Receiver operating characteristic
SIM: Subscriber identity module
SVM: Support vector machine
TN: True negative
TP: True positive
VoIP: Voice over internet protocol
WEKA: Waikato environment for knowledge analysis
Xgb: Extreme gradient boosting

## Declarations

### Clinical trial

Not applicable—this study is not a clinical trial.
